# Mapping out the scenarios of ocean energy scale-up based on the development of offshore wind

**DOI:** 10.12688/openreseurope.15906.1

**Published:** 2023-06-20

**Authors:** Anne-Caroline Pillet, Benjamin Lehner, Simon Stark, Hinne van der Zant

**Affiliations:** 1 Sup'Enr, Université de Perpignan Via Domitia, Perpignan, Occitanie, France; 2 Dutch Marine Energy Centre, The Hague, The Netherlands; 3 Universiteit Utrecht, Utrecht, The Netherlands

**Keywords:** Wave, Tidal, Offshore wind, European targets, Policy recommendations, Offshore energy

## Abstract

**Background:** Our oceans remain one of the last untapped source of renewable energy. The predictability and reliability of ocean energy technologies could contribute significantly to the global energy transition. By 2022, ocean energy, and in particular wave and tidal energy have reached a pre-commercial phase in their development.

**Methods:** This study investigates the potential progression of the wave and tidal energy sector in the next three decades based on the offshore wind sector in the past three decades. Two different models were developed from the yearly capacity increase of offshore wind in Europe and applied to the wave and tidal energy sector.

**Results:** According to both models, the 40 GW 2050 target for ocean energy set by the European Commission in 2020 could be reached if European coastal countries adopt supportive policies for both technologies immediately. A sensitivity analysis shows further that a small delay right now will have tremendous negative impacts to fulfill the EU goals and the contribution of ocean energy to the energy transition.

**Conclusions:** In conclusion, the ocean energy sector shows a strong growth potential and is capable of supporting the European and global climate targets substantially by 2050. Learnings from the offshore wind sector can help scope and support the growth of ocean energy technologies.

## Introduction

Ocean energy could contribute significantly to a reliable "Net Zero Emmission" energy system by 2050 ^1^. Wave and tidal energy are the two most advanced technologies in the ocean energy sector. Their predictability and reliability can help to improve grid forecasts and generally balance the grid to the demand. Also, the global resource potential for tidal and wave energy is around 30,700 TWh ^2^, which is higher than the total world-wide electricity consumption in 2019 ^3^.

In 2020, the European Commission has set three targets regarding the cumulative capacity of ocean energy commissioned: 100 MW in 2025, 1 GW in 2030 and 40 GW in 2050 ^4^. The European SET plan aims for a Levelized Cost of Energy (LCoE) of 100 EUR/MWh for tidal energy and 150 EUR/MWh for wave energy by 2030 ^5^. The capacity target and the LCoE target are highly interlinked and co-dependent. A higher installed capacity leads to cost reduction and performance improvements due to learning-rates and economy of scale. A lower LCoE in turn leads to more capacity installed due to a more competitive business-case.

By beginning of 2022 one-third of the time for the 2025 target has already passed but only 2% of the 100 MW goal in the EU have been reached. However, around 112 MW of tidal and wave power are in the project pipeline in European waters (excluding the United Kingdom) and could be deployed by 2025 ^6^. Moreover, according to Ocean Energy Europe (OEE) which used the Compound Annual Growth Rate (CAGR) methodology, between 1.5 GW (low growth scenario) and 2.88 GW (high growth scenario) of tidal and wave capacity could be deployed by 2030 ^7^. Regarding tidal power LCoE reduction, Offshore Renewable Energy Catapult (OREC) states that it could be around 181 EUR/MWh for a total of 100 MW installed, 108 EUR/MWh at 1 GW installed and 94 EUR/MWh at 2 GW installed ^8^.

The development of the ocean energy sector is accelerating according to the past year deployments and positive policy developments in the EU ^4^, US ^9^ and UK ^10^. Assuming that the wave and tidal energy sectors are on the brink of commercial feasibility, it is important to model the sectors potential contribution to the energy system in the next decades. To do so, the capacity growth of the more mature bottom-fixed offshore wind sector can be applied to the expected growth of ocean energy. The offshore wind and ocean energy sector share many comparable aspects regarding installation environment, required supply chain, operation and maintenance procedures and electrical infrastructure. The offshore wind sector has been growing exponentially in Europe since the 1990s. In 2021, almost 26 GW of offshore wind are commissioned and 100 GW more could be deployed by 2030 ^11^.

In this study, the three decades of offshore wind deployment from 1990 to 2020 are analysed to draw various forecast scenarios on the operating capacities and LCoE reduction for wave and tidal energy until 2050 in Europe. Based on the forecast of the development of the tidal and wave energy sectors the feasibility of meeting the European ocean energy targets regarding the capacity deployed and the LCoE is evaluated. The behaviour of the offshore wind market is further linked with policy support mechanisms showing the necessity of supportive policies for ocean energy technologies in European coastal countries.

## Methods

The wave and tidal energy sector development trajectories in this paper were based on the offshore wind growth in the past three decades. A database listing all the offshore wind farms in the pipeline, under-construction, commissioned and decommissioned within Europe was used. Only bottom-fixed offshore wind farms were considered as the offshore floating wind sector in 2022 was still at the early stage of commercial deployment. The first offshore wind array commissioned in Europe was named Vindeby and was installed off the coast of Denmark in 1991. The reference year for the offshore wind sector was consequently set to 1991. Both commissioned and decommissioned offshore wind farms were considered for the study.

In total, the data from 119 offshore wind farms was used to compute the results from eight different countries (BE, DE, DK, FI, IE, NL, SW, UK) from 1991 until 2021. An exponential growth curve and doubling time model was deducted from this dataset and applied to the wave and tidal energy sector. For both technology types the starting year and value equaling the offshore wind sector in 1991 were carefully assessed.

### Starting values and years

Between 2010 and 2021, 30.2 MW of tidal stream energy converters have been installed in Europe since 2010, of which 11.5 MW were in the water in 2021 and 12.7 MW of wave energy converters have been installed, of which 1.4 MW were in the water in 2021 ^12^. A database of all those deployments was used to define the starting year and value.

First, the status of the technology developers in the database was checked and companies who have since dissappeared or are hibernating for a prolonged time-period were filtered out. Second, the starting dates of entering the pre-commercial phase for both the wave and tidal sector were established based on publicly available data. The basic characteristics of the pre-commercial phase are: existence of Power Purchase Agreements (PPAs), first array installations, increased investor interest and policy support. Third, the starting year was matched with the cumulative capacity of still operating technology developers in that year resulting in the starting value.

For the tidal sector, 2016 was established as the year where the sector entered a pre-commercial phase. The dominating contribution came from Nova Innovation, Tocardo and the SIMEC Atlantis project (including tubines from Andritz Hydro). All three technology developers started to install the first turbines of larger-scale projects at that time. Together they had 3.05 MW installed which was considered the starting value for tidal energy in 2016. Additional active European technology developers who were not considered because they were in 2016 still more in a demonstration phase or deployed outside of Europe include Schottel Hydro, SME, Sabella, and ORPC.

For the wave sector, 2020 was established as the year where the sector entered a pre-commercial phase. In 2020 the constructions of the first MW scale systems started. Some prominent examples are the Corpower Ocean Wave Energy Converters array in Portugal, the Wello system in Spain and the full-scale demonstration of the Irish developer Ocean Energy in Hawaii. Others like EcoWavePower, Seabased and AW Energy demonstrated large-scale devices or arrays with power purchase agreements and showed a large project pipeline. Combined with several additional offshore tested technology developers a critical mass of wave energy companies has been reached. Altogether this means that the entrance into a pre-commercial stage started in 2020. Altogether those companies had a cummulative capacity of 6.11 MW in 2020, which was chosen as the wave model starting value ( Table 1).

**Table 1.** Tidal and wave developers taken into consideration to choose the starting values.

Tidal developers	Wave developers
Tocardo (NL)	40South Energy (IT)
Nova Innovation (UK)	AMOG (AU)
SIMEC Atlantis Energy (UK)	AW-Energy (FI)
	CorPower Ocean (SW)
	Crestwing (DK)
	Eco Wave Power (SW)
	Floating Power Plant (DK)
	Fred Olsen Ltd (UK)
	GEPS Techno (FR)
	Hace (FR)
	Havkraft AS (NO)
	Marine Power Systems (UK)
	Ocean Energy (IE)
	Resen Energy (DK)
	Seabased (IE)
	SINN Power (DE)
	Voith Hydro (DE)
	Wave for Energy (IT)
	Wavenergy (IT)
	Wavepiston (DK)
	Waves4Power (SW)
	Wedge (SP)
	Wello Oy (FI)

An important difference between offshore wind and some bottom-fixed tidal systems on the one side and wave energy devices on the other side is the ease by which the later can be deployed and towed back to shore for maintenance or improvements. Therefore, the cumulative capacity of companies still actively operating in the sector was used as the starting value for the wave energy sector, while only pre-commercial deployments were used for the tidal energy sector.

### Growth curve model

The first model - named "Growth curve model" - was developed based on an exponential fitting of the offshore wind cumulative capacity over the years. Firstly, the data was collected and only offshore wind farms commissioned and decommissioned between 1991 and 2021 in Europe with bottom-fixed foundations were taken into consideration. Secondly, the offshore wind turbine capacities were summed for each year and the decommissioned offshore wind turbines were subtracted whenever they have been decommissioned. Thirdly, a cumulative sum over the years was computed.

In order to apply the growth curve from the offshore wind sector to the ocean energy sector curve fitting was applied. The curve fitting was performed to achieve the highest correlation between the function and the real cuve, determined by the coefficient of determination *R* ^2^ values. The most appropriate function identified was an exponential split into three ten-year intervals (coefficients of determination between 0.915 and 0.993) : the "lag phase" from 1991 until 2001, the "kick-off phase" from 2001 until 2011 and the "growth phase" from 2011 until 2021 (and still going). If no intervals were considered an even higher average coefficient of determination was achieved (0.988). This, however, showed bad correlation with the datapoints in early years with low cumulative capacity. While the small values only led to small total derivation, the difference to what was really deployed was a factor 10 in the first 10 year interval (namely the "lag phase"). While this divergence did not weigh strongly for the overall correlation calculation it had a too strong influence on the prediction of the early growth of the sector.

A larger number of intervals led to a better fit to the data of offshore wind deployments, but it also led to undesired effects. Especially as specific local events in the offshore wind energy sector gained a too large weight in the curve fitting. This was not in line with the purpose of predicting a European growth trend for ocean energy.

The exponential coefficients of the growth curve were obtained by using the *curve_fit* Python function, applied to the following equation:


$$P_{cum}=a*e^{b*t_{y}}+P_{cum,0}-a*e^{b*t_{y,0}}\;(1)$$

where *a* and *b* are the two coefficients computed by the *curve_fit* function, *P _cum_* the cumulative capacity in MW, *t _y_* the number of years since the year of reference, *P*_*y*,0_ and *t*_*y*,0_ the first values of the interval.

The coefficients of the three exponential functions were then applied to the wave and tidal energy sector. Depending on the starting point, a 30-year period was not always sufficient to reach 2050. In that case, the remaining years were extrapolated with the exponential coefficients of the last interval.

The coefficient of determination *R* ^2^ was computed using the following equation:


$$R^{2}=1-\frac{\sum_{i}{\left(y_{dataset,i}-y_{fitting,i}\right)}^{2}}{\sum_{i}{\left(y_{dataset,i}-{\bar{y}}_{dataset}\right)}^{2}}\;(2)$$

where *y _dataset,i_* is the offshore wind cumulative capacity (from the dataset), *ȳ _dataset_* is the mean of the offshore wind cumulative capacities for each interval and *y _fitting,i_* is the offshore wind cumulative capacity given by the model. The closer *R* ^2^ approaches 1 the better the correlation between the dataset and the applied function. *R* ^2^ values above 0.9 are acceptable.

### Doubling time model

The second model was based on the number of doubling events during the 30-year period. A doubling event is defined as the doubling of the installed capacity of the renewable energy technology globally or in a certain area. The doubling event computation is frequently used when analysing the behaviour of a sector. In the renewable energy sector, it is typically used for learning rates but it can also be useful for other techno-economic calculations.

The doubling time method is equivalent to the Compound Annual Growth Rate method which is used in other studies forecasting the growth of the ocean energy sector ^7^.

Not only the number of doublings were computed but also the length of time between two doublings. These values gave the opportunity to have a more relatable idea of how a sector is growing. It was computed using the following equation:


$$\;T_{d}=(t_{2}-t_{1})*\frac{ln2}{ln\frac{q_{2}}{q_{1}}}\;(3)$$

where, *T _d_* is the length of time between two doublings, *t*_1_ the first year of the interval, *t*_2_ the last year of the interval, *q*_1_ the cumulative capacity for the first year of the interval in MW and *q*_2_ the cumulative capacity for the last year of the interval in MW.

The 30-year period was again split into six five-year intervals to have a sufficiently high coefficient of determination ( *R* ^2^ = 0.995). A five-year interval was chosen and the growth rate inside the interval was assumed constant. The values used to compute the number of doublings were the same as for the growth curve scenario, i.e. the cumulative capacity over the years of the European bottom-fixed offshore wind farms, minus the decommissioned offshore wind turbines.

The number of doublings were computed using the following equation:


$$\;N=\frac{ln\frac{q_{2}}{q_{1}}}{ln2}\;(4)$$

where *N* is the number of doublings, *q*_1_ the cumulative capacity for the first year of the interval in MW, *q*_2_ the cumulative capacity for the last year of the interval in MW.

The number of doublings from the offshore wind sector was applied to the ocean energy sector using the following equation:


$$\;q=q_{0}*2^{N}\;(5)$$

where *q* is the new value of the cumulative capacity for the last year of the interval and *q*_0_ the cumulative capacity value for the first year of the interval.

Between the two boundaries of the 5-year interval, the growth rate was assumed constant and the curve followed an exponential growth following the equation displayed below:


$$\;q(t)=q_{0}*2^{\frac{t}{T_{D}}}=q_{0}*e^{r*t}\;(6)$$

where *q*( *t*) is the cumulative capacity after a timeinterval t, *q*_0_ is the cumulative capacity for the first year of the interval, *T _d_* the length of time between two doublings (known thanks to the offshore wind sector computing), *r* the constant growth rate and *t* the length of time in years.

Similar to the growth curve model and depending on the starting point, a 30-year period was not always enough to reach 2050. In that case, the remaining years were extrapolated with the average of the number of doublings for the last three intervals known.

**Figure 1.**
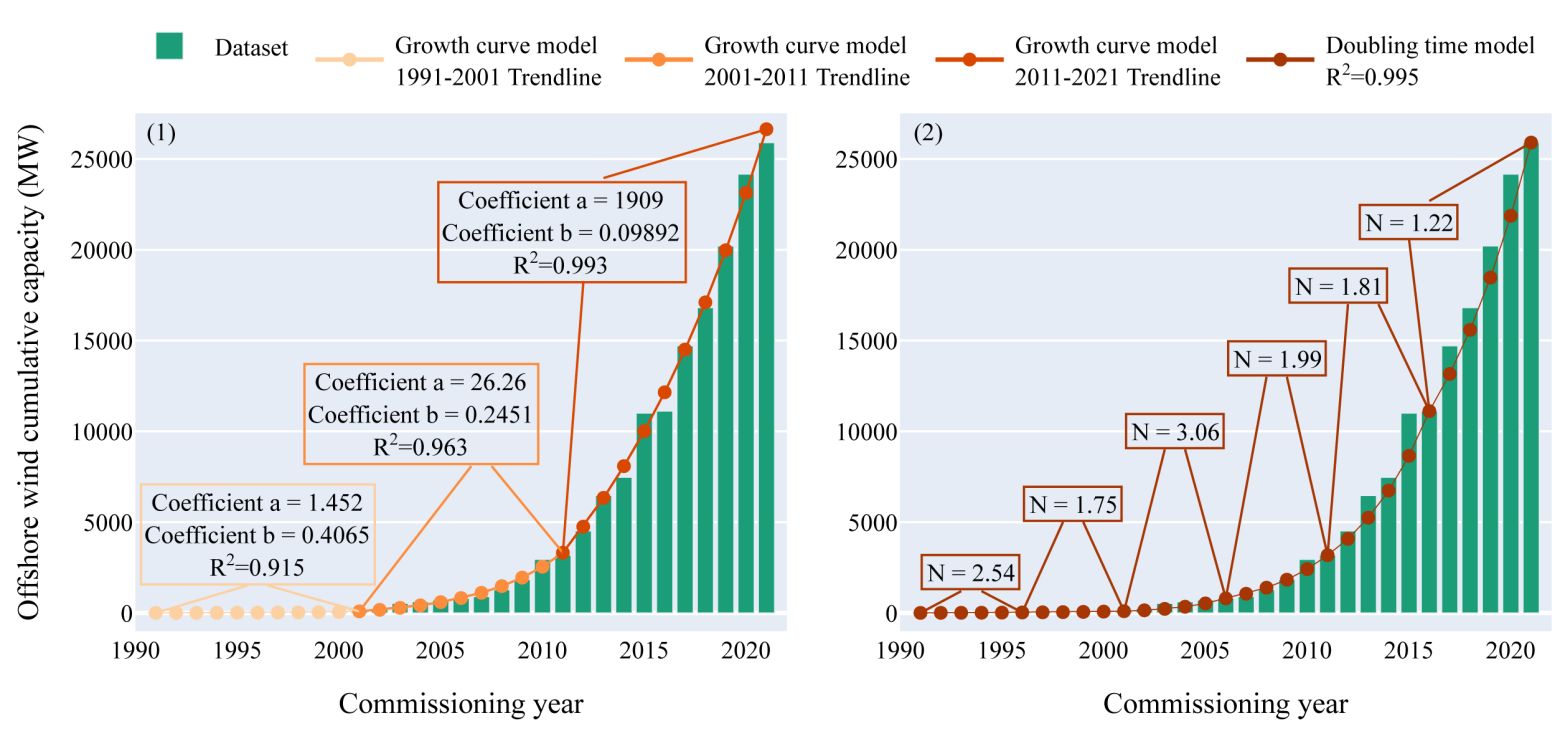
Offshore wind cumulative capacity over the commissioning years. ( **1**) Growth curve model. The fitting curve is split into three ten-year intervals: the "lag phase", the "kick-off phase" and the "growth phase". The coefficient of determination is higher than 0.9 for the three intervals. ( **2**) Doubling time model. The whole period is split into six five-year intervals in order to follow the original dataset closely. The global value of the coefficient of determination is higher than 0.99 which indicates that the model is close to the original dataset.

The real offshore wind cumulative capacity and the trendlines from both models are displayed in Figure 1. The coefficients of the trendlines and the number of doublings displayed within the figure were then used to compute the forecasts for the ocean energy sector.

The coefficients of determination *R* ^2^ for the exponential fittings of the offshore wind cumulative capacity in the growth curve model and the doubling time model were all above 0.9 (and even higher than 0.95 for two intervals of the growth curve fitting and the doubling time fitting) which means that both models were close to the original dataset.

### Levelized Cost of Energy forecast

In addition, the growth model for wave and tidal energy were combined with the LCoE forecast of OEE based on an ORE Catapult analysis ^7, 8^ ( 2). The LCoEs were computed depending on their capacity which was aligned with the expected commissioning year. The OEE dataset covers the LCoE for a cumulative capacity between 1 MW and 2,000 MW, any year with higher capacities then 2,000 MW was therefore not considered.

**Table 2.** LCoE forecast from OEE.

Cumulative capacity (MW)	Tidal LCoE (€/MWh)	Wave LCoE (€/MWh)
1	616	702
5	489	546
10	361	387
20	267	269
50	214	207
100	181	168
200	154	136
500	126	110
1,000	108	92
2,000	94	81

These data points have been transferred into a curve by linearly interpolating between the values given in the table. The cumulative capacity expected according to both models for each year between 1 MW and 2,000 MW are then compared to the curve to spot when there are matches between the LCoE forecast and our models. The LCoE given by the curve is then the one that can be expected for the commissioning year that goes with the cumulative capacity.

## Results and analysis

**Figure 2.**
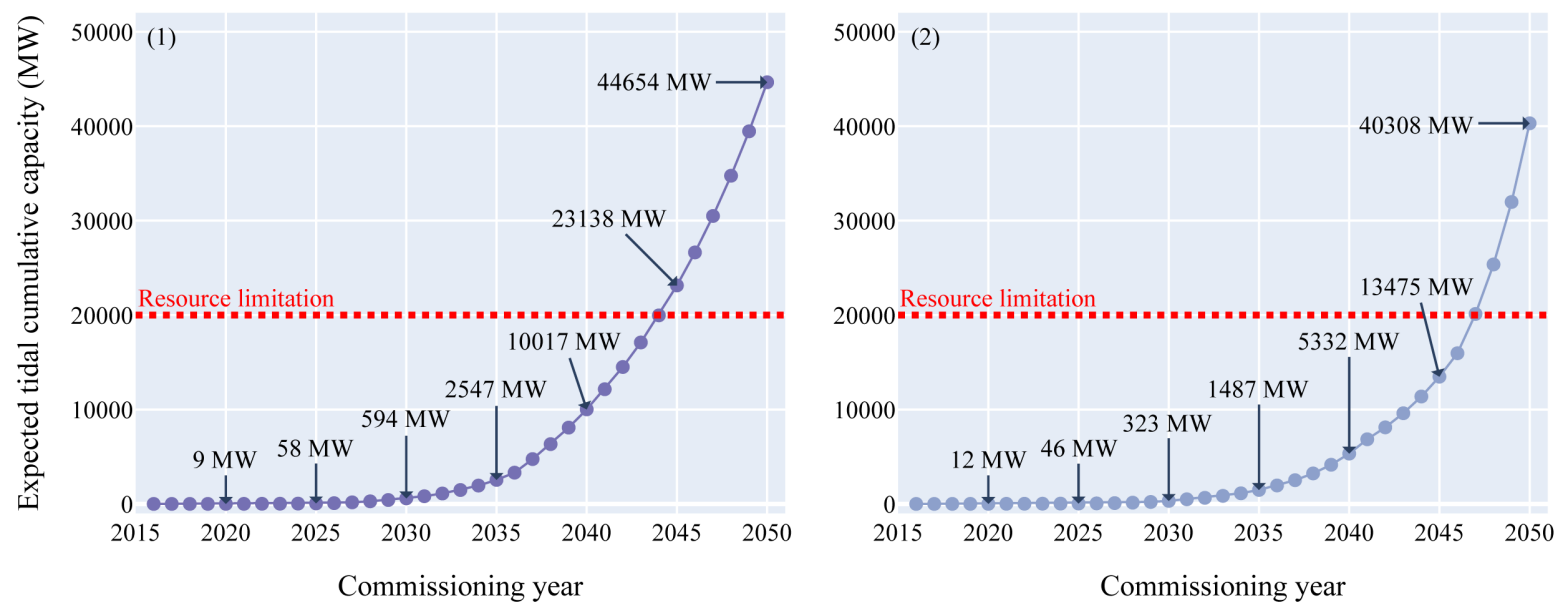
Expected tidal cumulative capacity over the commissioning years. ( **1**) Growth curve model - ( **2**) Doubling time model. Both models returned values with the same order of magnitude: between 46 and 58 MW could be deployed in 2025, between 323 and 594 MW in 2030 and between 40.3 and 44.7 GW in 2050. The red line displayed on the figure represents the tidal energy technical potential limitation in Europe which is around 20 GW. This limitation will be reached between 2044 and 2047 according to the models.

**Figure 3.**
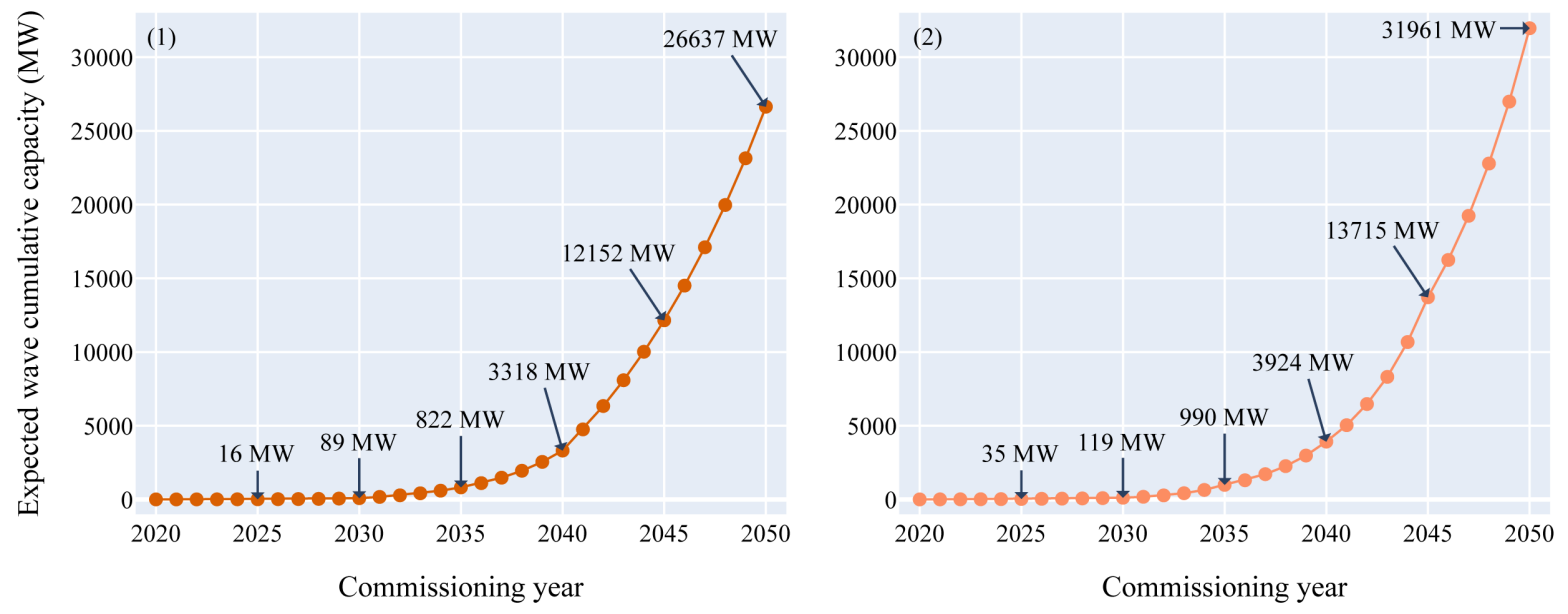
Expected wave cumulative capacity over the commissioning years. - ( **1**) Growth curve model - ( **2**) Doubling time model. According to both models, between 16 and 35 MW of wave power could hit the water by 2025, between 89 and 119 MW by 2030 and between 26 and 32 GW by 2050. As the wave energy technical potential in Europe is not expected to be reached by 2050, the sector will likely keep growing significantly after 2050. The wave energy resource potential limitation (around 100 GW) is outside the graph boundaries and will not be met by 2050 according to both models.

In order to understand the growth of the ocean energy sector in the next three decades the exponential growth and doubling time function were established for offshore wind ( Figure 1) and applied to the ocean energy sector. In 2016 and 2020 the starting capacity for tidal energy was estimated to 3.05 MW and the one of wave energy to 6.11 MW, respectively. Combining the starting capacities and years of wave and tidal with the exponential growth curve of offshore wind resulted in two growth curves per technology ( Figure 2, Figure 3).

The tidal energy sector entered its pre-commercial phase in 2016. Therefore, the amount of capacity that could be commissioned in the upcoming years according to the models is higher than for wave energy. The analysis showed that around 50 MW in 2025 (half of the European target) and between 320 and 600 MW in 2030 would be achievable. Between 2040 and 2044, 10 GW could be deployed and in 2050 between 40.3 and 44.7 GW of tidal power could theoretically be reached.

The wave energy sector reached its pre-commercial phase about four years after the tidal energy sector in 2020. Therefore, the deployed capacities in the upcoming years are lower than for tidal. In 2025, between 16 and 35 MW could be commissioned and between 89 and 119 MW in 2030. According to both models, 10 GW could be in the water in 2044. In 2050, between 26 and 32 GW of wave power could be deployed in Europe.

### Assessment of the resource potential

The results of the model were compared to the energy resource potentials to verify if the results are realistic. Depending on the technologies used to produce electricity, the efficiency varies. Therefore, there is a difference between the theoretical resource potential, which covers the total extractable amount of energy, and the technical resource potential, which includes limitations of current technologies.

For tidal energy, the technical resource potential limitation taken into consideration for this study is 20 GW ^8, 13^.

The 20 GW limitation for tidal cumulative capacity could be reached in 2044 for the growth curve scenario and in 2047 for the doubling time scenario.

For wave energy, the technical energy potential in Europe is around 95 GW (Schlütter *et al.* ^14^). According to both models, this limitation is not expected to be reached by 2050. Therefore, the expected cumulative capacity for tidal and wave energy combined could reach between 46.6 and 52 GW in 2050.

### European targets

According to the models, wave and tidal capacity will be crucial to meet the 100 MW target by 2025 set by the European Union. The remaining capacity (less than 30 MW) could be partly filled with salinity gradient power plants, OTEC power plants or floating solar modules.

**Figure 4.**
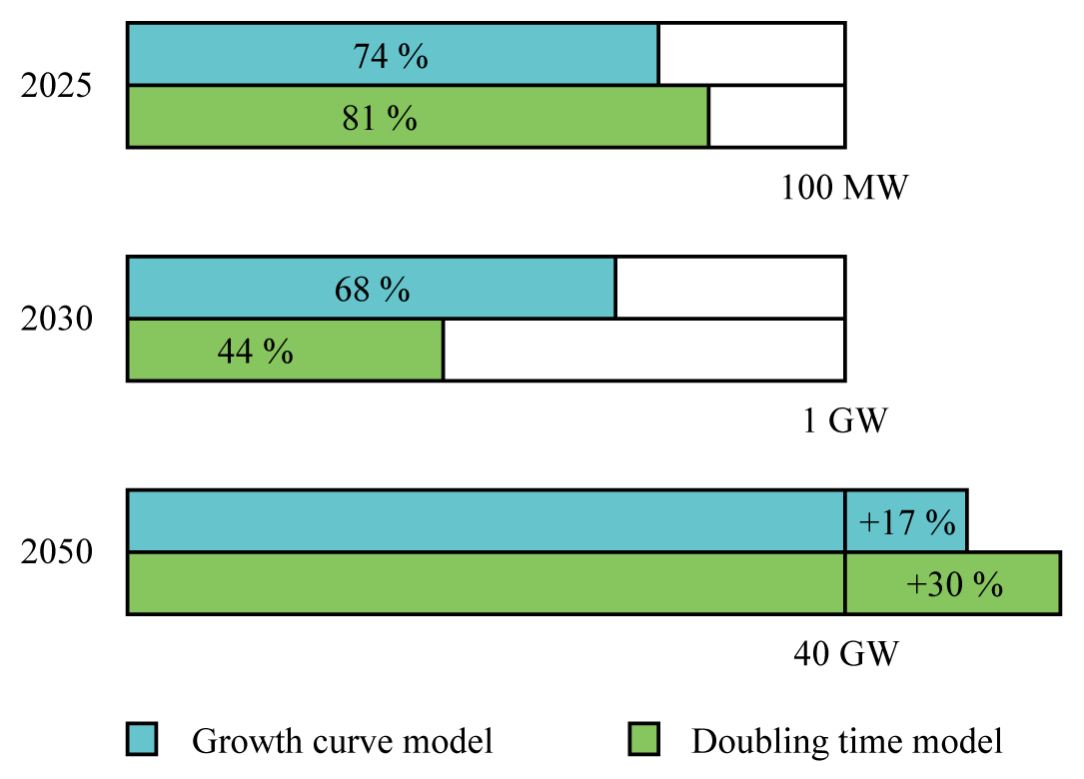
Completion percentage of the European targets. According to both models, the 2025 and 2030 targets will not be reached. Yet, the 2050 target could be overtaken. In order to get closer to the 2025 and 2030 targets, intensive support from European coastal countries are needed.

In the same way, the 2030 target of 1 GW will not be reached. Depending on the model considered, between 44 % and 68 % of the target could be filled with wave and tidal energy ( Figure 4). The remaining capacities would need to come from other ocean energy technologies.

According to both models, the 40 GW target by 2050 is expected to be overtaken by 6 to 12 GW. Furthermore, adding floating solar, salinity gradient and OTEC capacities, the 2050 target can be even further overtaken. Important to note is that the UK was considered to contribute to the European targets.

### Levelized Cost of Energy forecast

**Figure 5.**
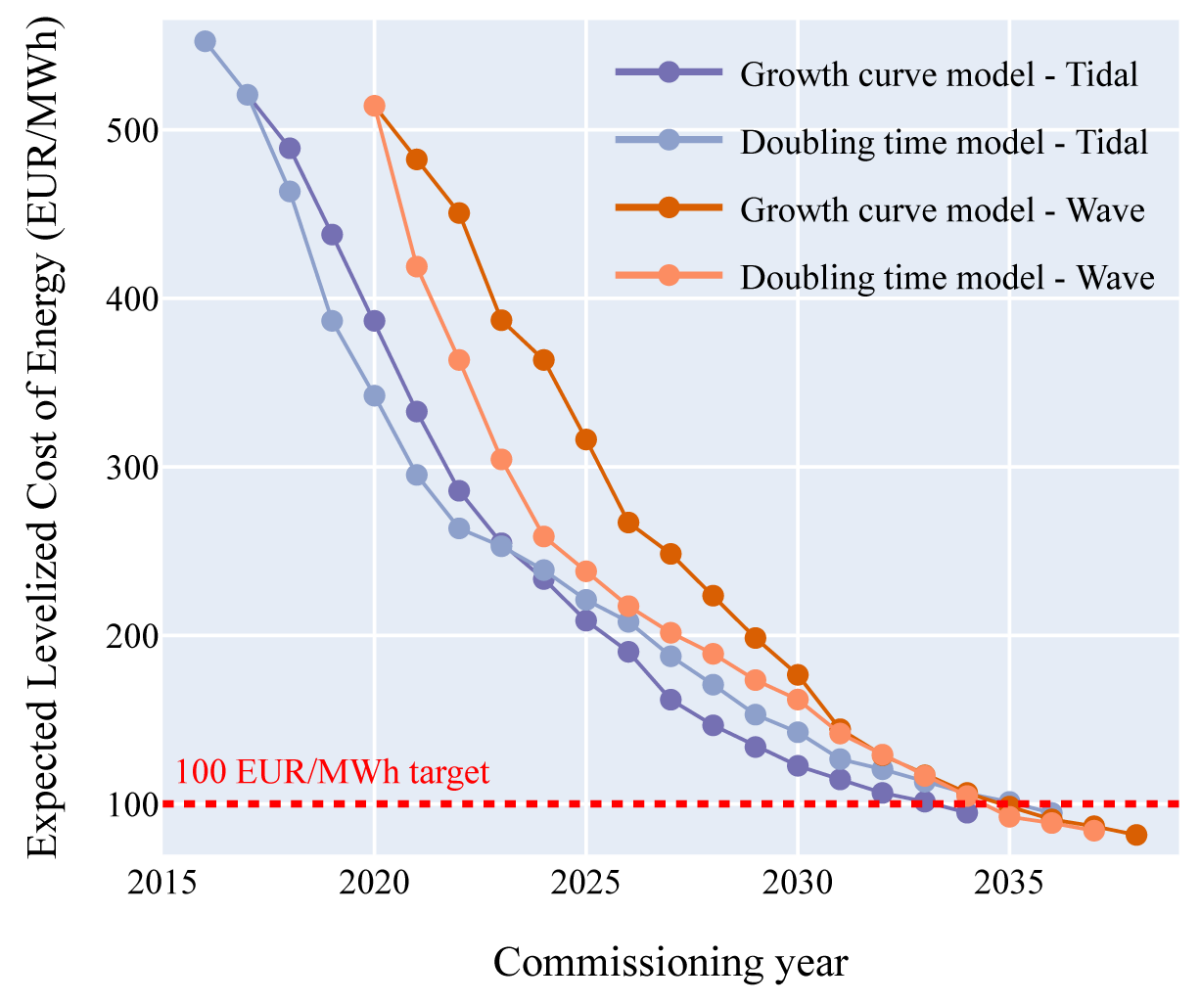
Expected LCoE over the commissioning years (the OEE dataset give the LCoE between 1 MW and 2000 MW of cumulative capacity). The 100 EUR/MWh target set by the European Commission could be reached, for both technologies, around 2035. The wave LCoE is higher than the tidal LCoE for the first capacities deployed but as more capacities hit the water, the LCoE for both technologies comes closer together.

Following the methodology given above, the expected LCoE over the commissioning years for each model and each technology are computed ( Figure 5).

It is generally accepted that a five-year delay between tidal LCoE and wave LCoE will occur ^5^. Yet, according to the models, the delay should be around five years during the first commercial deployment phase and then could shorten gradually until 2035 where the LCoE for both technologies will likely be equivalent.

The European Commission set targets for wave and tidal energy LCoE in the SET Plan ^5^: 100 EUR/MWh for tidal energy and 150 EUR/MWh for wave energy by 2030. The targets for 2030 will be sligtly delayed. According to both models, the 100 EUR/MWh target for tidal energy could be reached between 2033 and 2035 and the 150 EUR/MWh target for wave energy could be met in 2031. The forecasts given by OEE and in this study show consistency with the newest developments in tidal energy. In particular the award of 41 MW of tidal energy in the UK at a strike price of 208 EUR/MWH aligns well with the predicted 214 EUR/MWh for 50 MW ^15^.

### Sensitivity analysis of both models

**Figure 6.**
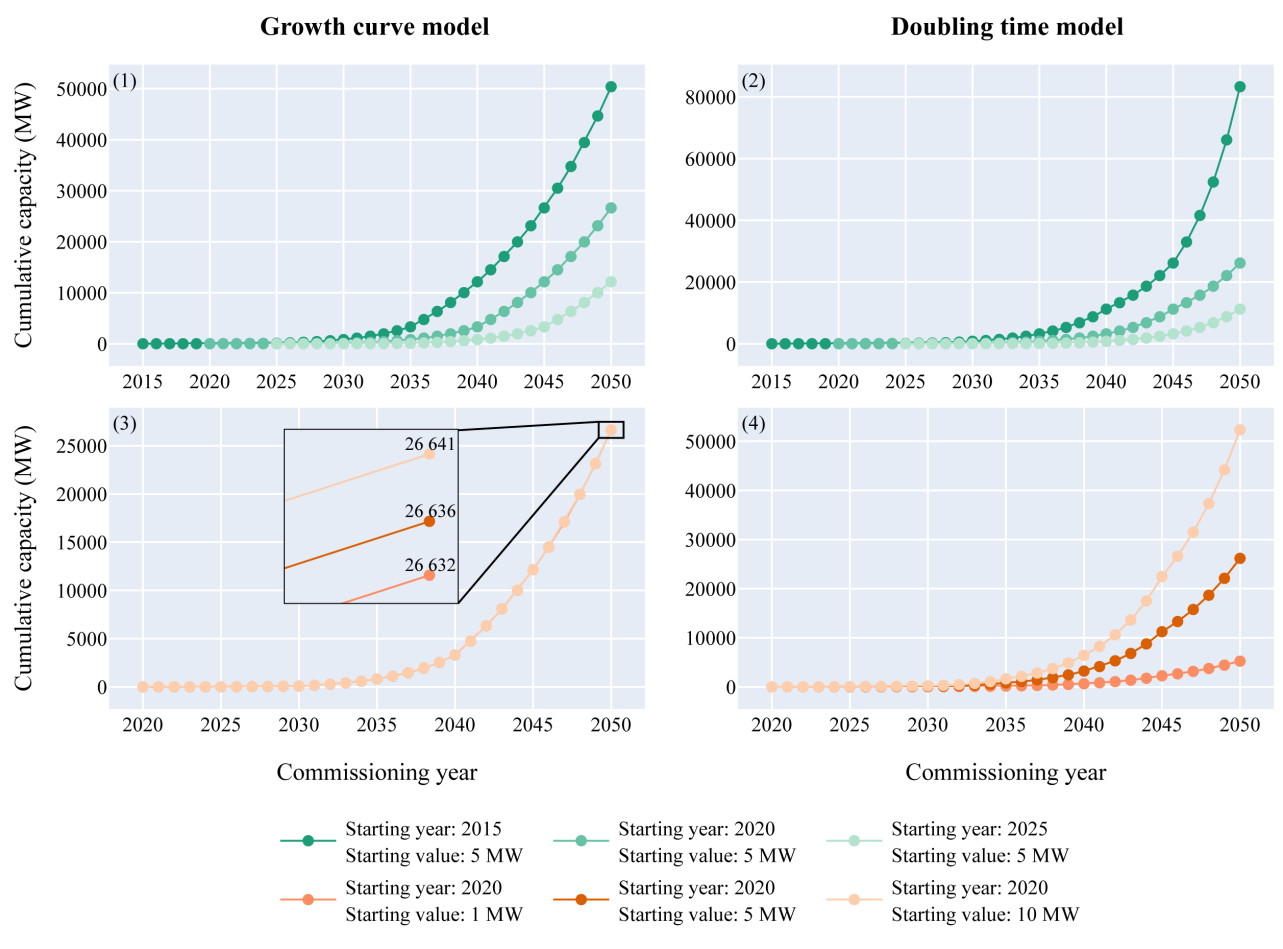
Sensitivity analysis of both models. ( **1**) Growth curve model, varying parameter=starting year - ( **2**) Doubling time model, varying parameter=starting year - ( **3**) Growth curve model, varying parameter=starting value - ( **4**) Doubling time model, varying parameter=starting value.

A sensitivity analysis was conducted for both models to understand how the models react when the starting year and the starting value vary ( Figure 6). Three different starting values (1 MW, 5 MW and 10 MW) and three different starting years (2015, 2020, 2025) were computed.

The variation of the starting year moves the curve to the left or to the right for both models. Therefore, as the growth is exponential, the expected capacity in 2050 changes drastically. For the growth curve model, if the starting year happens five years before, than the operating capacity in 2050 would be multiplied by 2.2. On the contrary, if the starting year happens five years later, than the operating capacity would almost be reduced by half. Regarding the doubling time model, the variation is even higher. A decrease of five years in the starting year leads to a 3.2 times higher capacity and an increase of five years leads to a 2.3 times lower capacity. The variation of the starting value does not have a significant effect for the growth curve model as it is only moving the curve up or down by the difference of the starting capacity. Therefore, a 5 MW variation of the starting value leads to a 5 MW difference in 2050, which is insignificant compared to the GW scale at this point. For the doubling time model, the variation of the starting value has a significant effect. If the starting capacity is doubled, then in 2050 the expected capacity will be doubled too. The starting year and the starting value are intrinsically linked but this analysis clearly highlights that a few years delay in the start of the commercial journey will significantly influence the operating capacities in 2050.

### Offshore wind support schemes

**Figure 7.**
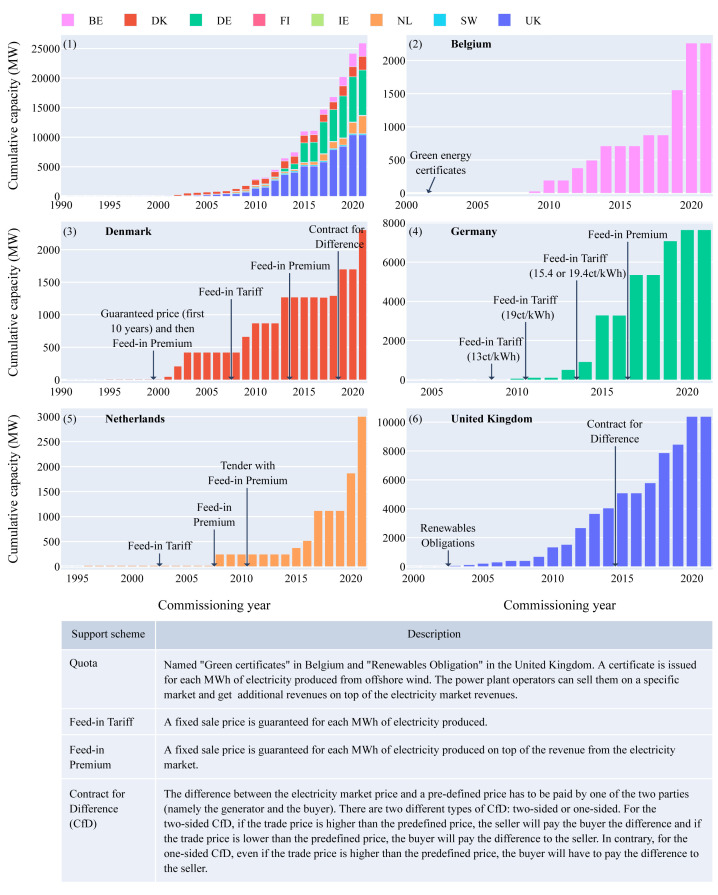
( **1**) Offshore wind cumulative capacity over the years for each European country - ( **2**) Offshore wind support schemes in Belgium - ( **3**) Offshore wind support schemes in Denmark - ( **4**) Offshore wind support schemes in Germany - ( **5**) Offshore wind support schemes in the Netherlands - ( **6**) Offshore wind support schemes in the United Kingdom. The first figure highlights the five European leading countries for offshore wind in terms of capacity deployed. Four different support schemes were used during the past 20 years: Feed-in Tariff, Feed-in Premium, Contract for Difference and Quota.

In 2022, five countries lead the European offshore wind market: Belgium, Denmark, Germany, the Netherlands and the United Kingdom with various offshore wind support mechanisms ( Figure 7).

The average length of time between the awarding of an offshore wind farm and its commissioning is around five years ^16^. Therefore, the effect on capacity installed of new support schemes only materializes five years after. To reach the European targets for ocean energy but also for offshore wind, shorter commissioning times are required. This result is consistent with the 5-year delay between the establishment of a new support scheme and the commissioning of offshore wind farms in various European countries ( Figure 7). Therefore the ocean energy sector does not only need subsidies but also needs to reduce the time needed for commissioning of new offshore farms. The latter can be accomplished by an already established offshore supply chain, usage of satellite data to shorten MetOcean campaigns, a detailed generally agreed on marine spatial plan and parallel permitting procedures instead of cascading ones taking all offshore renewables into consideration.

Similar to offshore wind, these subsidies will allow wave and tidal energy to mature and learn until they become profitable without direct subsidies from the government (e.g. Dutch offshore wind sector 2021). One way of structuring those different support schemes and subsidies in a transparent way ensuring the timely execution of projects, are offshore renewable tenders. Those tenders can specify what exactly the government supplies, what the boundaries are and what the goal of the tender is. A bidding system on those tenders increases competitiveness and value for money. An important indirect subsidy often interlinked with a tender structure is the provision of the offshore grid. This comes with the positive side-effect that the offshore grid will not only be optimized on the project developers profit, but also on energy security and overall system costs.

### Best case scenario

If an uncompromising policy support is set quickly to assist the tidal and wave energy sector growth, a very fast development of new or hibernating projects can be expected. It could also lead to a consolidation of technologies with a standardization of components used in wave and tidal devices. This standardization will further reduce the costs as supply chains become more competitive and reliable. Moreover, the tidal and wave sectors could benefit from the existing offshore supply chain developed for the offshore wind sector.

By introducing support schemes to support the sector immediately the "lag phase" that occured for the offshore wind sector in the 1990s is avoided and the 2025 and 2030 European targets can be met as well.

**Figure 8.**
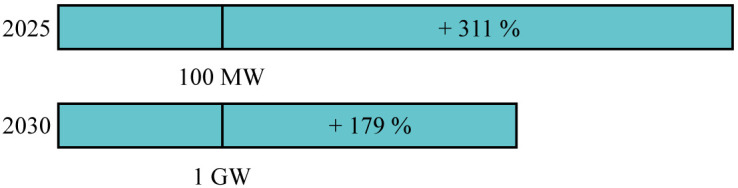
Completion percentage of the European targets in the best case scenario using the growth curve model. The European targets could be greatly overtaken if actions are taken now.

**Figure 9.**
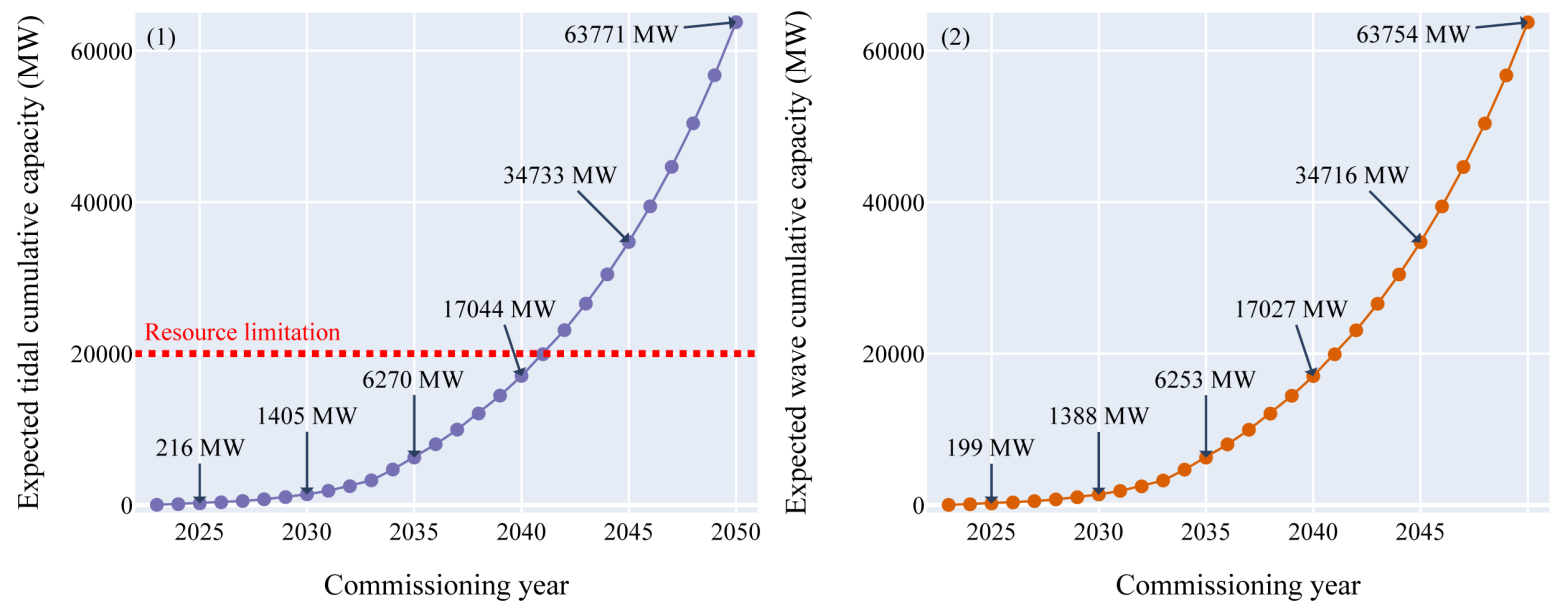
Expected tidal and wave cumulative capacities according to the best case scenario using the growth curve model. - ( **1**) Tidal - ( **2**) Wave.

The second phase of the offshore wind development started around 2001 with the introduction of support schemes. When computing the growth curve model using 2001 as the new starting year for offshore wind and 2023 as the starting year for both the tidal energy sector and the wave energy sector (see Appendix, Figure 9), the 2025 and 2030 European targets regarding the operating capacity could be greatly overtaken ( Figure 8). The starting values taken into consideration are 26.58 MW for the tidal sector and 9.57 MW for the wave sector. These values are obtained by computing the growth curve model using the initial starting points and considering the expected cumulative capacity in 2023.

## Discussion and conclusion

A huge amount of untapped energy is located in our oceans and seas. Ocean energy technologies like wave and tidal are needed to tap this last big reservoir of renewable enery and are needed to meet a global "Net Zero Emission" energy system. Europe is leading the ocean energy sector but the US and China are also catching up. Only by a fast implementation of strong support policies to further develop it, Europe will stay on top generating unique export opportunities. Doing so and taking all limitations into consideration it is likely that both the wave and tidal sector will grow in a similar fashion than offshore wind. In the last three decades, more than 25 GW of offshore wind was commissioned in European waters. This study, based on the development of the offshore wind sector, forecasts around 50 GW of wave and tidal operating capacity in Europe by 2050.

According to the models developed for this study, the full technical potential of 20 GW of tidal energy could be deployed between 2044 and 2047. Following the offshore wind growth, intermediate values between 46 and 58 MW in 2025 and between 323 and 594 MW in 2030 are realisticly achievable .

Regarding the wave operating capacity, between 26 and 32 GW could be deployed by 2050. As the resource potential in Europe for wave energy is far higher than that, further growth after 2050 is likely. Moreover, between 16 and 35 MW of wave energy could be deployed by 2025 and between 89 and 119 MW by 2030.

According to the models and based on the limited policy commitments of European coastal countries to this date, the European targets set for 2025 and 2030 (respectively 100 MW and 1 GW of ocean energy) will not be met. In total, between 74 and 81 MW of wave and tidal energy could be deployed by 2025 and between 442 and 683 by 2030. Other ocean energy technologies such as OTEC and salinity gradient will unlikely be enough to fill the gap in those years. In 2050, taking into consideration the tidal resource limitation, between 46.6 GW and 52 GW could be deployed, greatly overtaking the European target of 40 GW.

Based on the results from both models and the LCoE forecasts from OEE, the 100 EUR/MWh LCoE target for tidal energy could be met in 2033 and the one of wave energy in 2035. This cost reduction makes the sector cost competitive to other energy sources. However, considering the international developments in 2022 and the tremendously increased cost of electricity one could argue that those technologies are already now price competitive if being installed in arrays.

The main assumption of this study is that the tidal and wave energy sectors will behave the same way as the offshore wind sector did in the past. But if we want the ocean energy sector to grow the same way than the offshore wind sector, we need to be sure that the ocean energy sector benefits from the same or, given the time constraint at hand, even better support from European coastal countries. Moreover, the sooner the supportive measures will occure, the higher the operating capacity will be. As seen with the sensitivity analysis, a five-year delay can lead to almost a 50 % decrease in the operating capacity in 2050.

The growing of the offshore wind sector should be taken as an example in terms of support schemes, but the ocean energy sector can learn from previous misconceptions. In particular a reduction of delays between the establishment of a policy and when it is applied to a project and the increased speed of permitting and consenting will be critical. At the European level and for some European countries, the first positive developments are visible (European ocean energy targets, European initiative to standardize and fasten permitting procedures, UK’s contract for difference on tidal energy, commitments of the Portuguese and Spanish government, etc.). Overall, the ocean energy sector shows great potential to support the European and global climate targets.

## Data Availability

Zenodo. European offshore wind farms and ocean energy deployements. DOI: 10.5281/zenodo.7938413. This project contains the following underlying data: Offshore_wind_farm_european_deployements.xlsx (European offshore wind farms dataset used to forecast the development of ocean energy in Europe in the upcoming three decades). Tidal_Energy_Converters_European_deployements.xlsx (Tidal energy converter deployments in Europe dataset used to forecast the development of ocean energy in Europe in the upcoming three decades). Wave_Energy_Converters_European_deployements.xlsx (Wave energy converter deployments in Europe dataset used to forecast the development of ocean energy in Europe in the upcoming three decades) Data are available under the terms of the Creative Commons Attribution 4.0 International license (CC-BY 4.0).
