# Mapping out the scenarios of ocean energy scale-up based on the development of offshore wind

**DOI:** 10.12688/openreseurope.15906.2

**Published:** 2025-01-28

**Authors:** Anne-Caroline Pillet, Benjamin Lehner, Simon Stark, Hinne van der Zant

**Affiliations:** 1 Sup'Enr, Université de Perpignan Via Domitia, Perpignan, Occitanie, France; 2 Dutch Marine Energy Centre, The Hague, The Netherlands; 3 Universiteit Utrecht, Utrecht, The Netherlands

**Keywords:** Wave, Tidal, Offshore wind, European targets, Policy recommendations, Offshore energy

## Abstract

**Background:**

Our oceans remain one of the last untapped large sources of renewable energy. The predictability and reliability of marine energy technologies could contribute significantly to the global energy transition. By 2022, marine energy, and in particular wave and tidal energy have reached a pre-commercial phase in their development.

**Methods:**

This study investigates the potential progression of the wave and tidal energy sector in the next three decades based on the offshore wind sector in the past three decades. Two different models were developed from the yearly capacity increase of offshore wind in Europe and applied to the wave and tidal energy sector.

**Results:**

According to both models, the 40 GW 2050 target for marine energy set by the European Commission in 2020 could be reached if European coastal countries, including countries associated to the EU-27, adopt supportive policies for both technologies immediately. A sensitivity analysis shows further that a small delay right now will have tremendous negative impacts on fulfilling the EU goals and the contribution of marine energy to the energy transition.

**Conclusions:**

The ocean energy sector shows a strong growth potential and is capable of supporting the European and global climate targets substantially by 2050. Lessons learned from the offshore wind sector can help scope and support the growth of marine energy technologies.

## Introduction

Offshore renewable energy will contribute significantly to a reliable "Net Zero Emission" energy system by 2050 ^1^. The offshore energy transition is currently led by wind energy, but wave and tidal energy are also advancing fast. Those two are the most advanced technologies in the ocean energy sector which includes next to wave and tidal energy also ocean thermal energy conversion (OTEC) and salinity grandient energy. The predictability ^2^ and reliability of these technologies can help to improve grid forecasts and generally balance the grid to the demand ^3^. Moreover, numerous studies have described synergies of a combined wave-wind park, including enhancing cost competitiveness of wave energy ^4– 6^. The global theoretical resource potential for tidal, wave and wind energy is 1,200 ^7^, 18,500 ^8^ to 29,500 ^7^ and 36,000 ^9^ TWh/year respectively. All three offshore renewable energy (ORE) sources together have a higher potential than the total world-wide electricity consumption in 2019 ^10^.

Enabling this high potential is dependent on the techno-economic feasibility commonly measured via the Levelized Cost of Energy (LCoE) in comparison to the average day-ahead-electricity price. The average day-ahead electricity price is on a constant rise over the last decade and, fueled by the Ukrainian war, increased sevenfold between 2020 and 2022 to 235 €/MWh ^11^. The LCOE of wind energy, tidal energy and wave energy was, in 2022, 81 EUR/MWh ^12^, 207 EUR/MWh ^13^ and 258 €/MWh ^14^, respectively. The targets established in the European SET plan aim for a LCoE of 100 EUR/MWh for tidal energy and 150 EUR/MWh for wave energy in 2030 ^15^. It is important to look at the LCoE in correlation with the planned capacity targets in Europe as both are highly interlinked and co-dependent. A higher installed capacity leads to cost reduction and performance improvements due to learning-rates and economy of scale. A lower LCoE in turn leads to more capacity installed due to a more competitive business-case. In 2020, the European Commission has set three targets regarding the cumulative capacity of ocean energy (100 MW in 2025, 1 GW in 2030 and 40 GW in 2050) and offshore wind energy commissioned (60 GW in 2030 and 300 GW in 2050) ^16^.

While by the beginning of 2022 33% of the time for the EU 2025 target has already passed, only 2% of the 100 MW capacity goal have been reached. However, around 112 MW of tidal and wave power are in the project pipeline in EU-27 waters (excluding affiliate countries) and could be deployed by 2025 ^17^. Next to these policy targets also research has been published by different European sector organisations and research centres. According to Ocean Energy Europe (OEE) which uses the Compound Annual Growth Rate (CAGR) methodology, between 1.5 GW (low growth scenario) and 2.88 GW (high growth scenario) of tidal and wave capacity could be deployed by 2030 ^18^. The Offshore Renewable Energy Catapult (OREC) found that LCoEs could be around 181 EUR/MWh for a total of 100 MW installed, 108 EUR/MWh at 1 GW installed and 94 EUR/MWh at 2 GW installed ^19^.

The development of the ocean energy sector is accelerating due to positive policy developments in the ranging from the innovation fund and Horizon Europe program in the EU ^20^, the Inflation Reduction Act with clear commitment to ORE in the US ^21^ and market pull frameworks like the contract for difference on tidal energy in the UK ^22^. Japan ^23^, China ^24^ and the Republic of Korea ^25^ are already advancing their offshore wind industries and are also investigating how wave and tidal energy can complement their energy mix.

Assuming that the wave and tidal energy sectors are on the brink of commercial feasibility, it is important to establish the sector’s potential contribution to the energy system in the next decades The most commonly used energy system models (DNV ^26^, IEA ^27^, etc.) are not yet including wave and tidal energy. Only a few, including the [R]Evolution scenario of Greenpeace which calculate the ocean energy capacity in OECD Europe by 2050 to be 53 GW ^28^ and the IRENA 1.5 degrees model, which aims for 351 GW worldwide by 2050 ^29^. To provide sufficient data to have more energy system reports including ocean energy in a reliable way, the capacity growth of the more mature bottom-fixed offshore wind sector can be applied to the expected growth of wave and tidal energy. These ORE sectors share many comparable aspects regarding installation environment, required supply chain, operation and maintenance procedures and electrical infrastructure. The offshore wind sector has been growing exponentially in Europe since the 1990s. In 2021, almost 26 GW of offshore wind are commissioned and 100 GW more could be deployed by 2030 ^30^.

In this study, the three decades of offshore wind deployment from 1990 to 2020 are analysed to draw various forecast scenarios on the operating capacities and LCoE reduction for wave and tidal energy until 2050 in Europe. Based on the forecast of the development of the tidal and wave energy sectors the feasibility of meeting the European ocean energy targets regarding the capacity deployed and the LCoE is evaluated. The behaviour of the offshore wind market is further linked with policy support mechanisms showing the necessity of supportive policies for ocean energy technologies in European coastal countries.

## Methods

**Figure 1.**
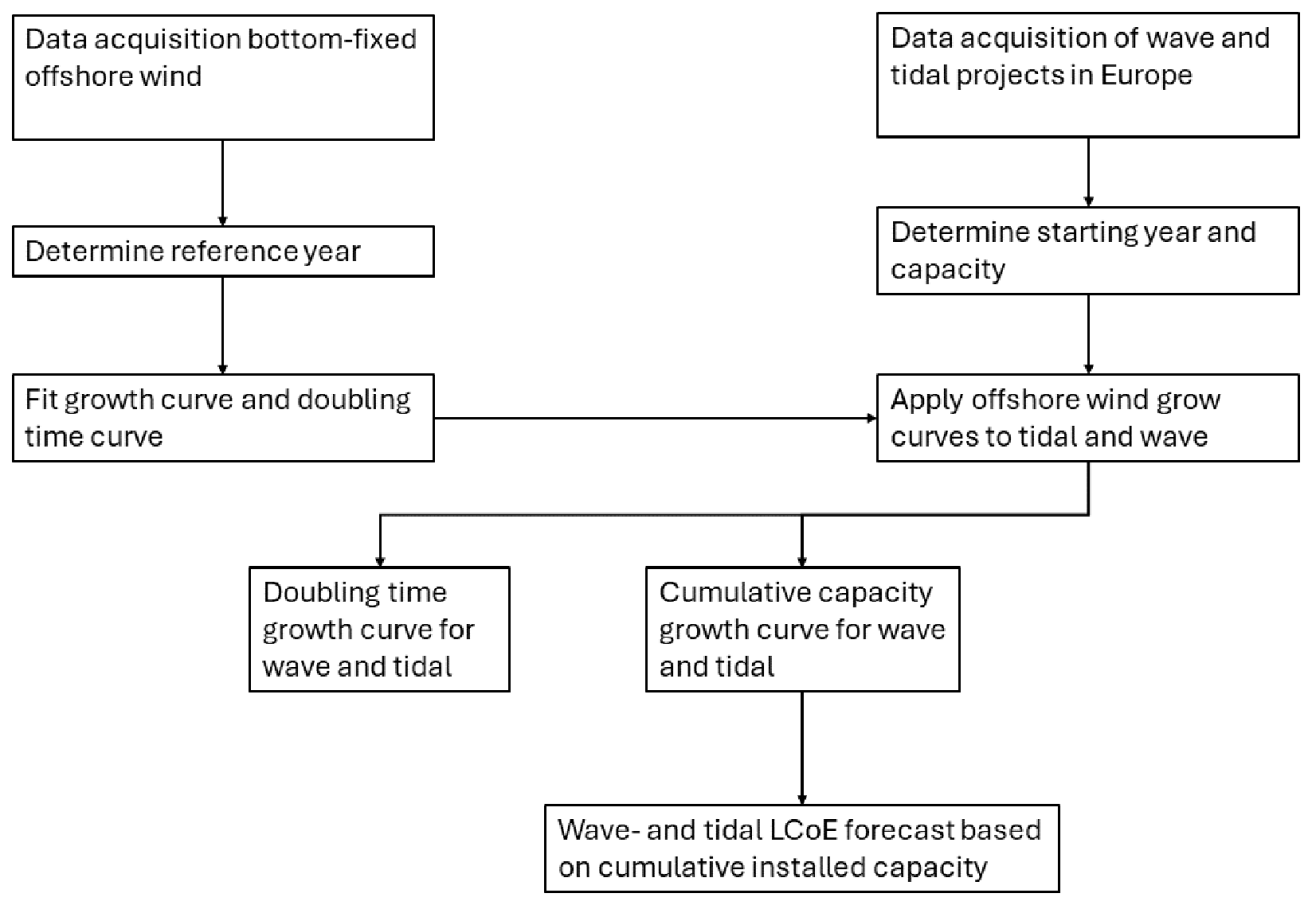
Figure 1 depicts a flowchart summarizing the key steps that were used to build the model presented in this paper. Each step will be further elaborated upon in this section.

Figure 1 presents a flowchart summarizing the methodological steps used to develop the cumulative growth curve and doubling time models for wave and tidal energy. It outlines the process from data acquisition to model development, detailing the determination of the reference year for offshore wind and the derivation of growth parameters based on deployment data from 1991–2021. These growth curves were adapted for tidal and wave energy by identifying their respective pre-commercial phases and establishing starting capacities. The flowchart provides a visual guide to these steps, which are elaborated upon in the following sections.

### Data acquisition

The wave and tidal energy sector development trajectories in this paper were based on the offshore wind growth in the past three decades. A database listing all the offshore wind farms in the pipeline, under-construction, commissioned and decommissioned within Europe was used (published along with this article). Only bottom-fixed offshore wind farms were considered as the offshore floating wind sector in 2022 was still at the early stage of commercial deployment. The first offshore wind array commissioned in Europe was named Vindeby and was installed off the coast of Denmark in 1991. Based on this first commercial array installation, the reference year for the offshore wind sector was consequently set to 1991. Both commissioned and decommissioned offshore wind farms were considered for the study.

In total, the data from 119 offshore wind farms was used to compute the results from eight different countries (BE, DE, DK, FI, IE, NL, SE, UK) from 1991 until 2021. An exponential growth curve and doubling time model was deducted from this dataset and applied to the wave and tidal energy sector. For both technology types the starting year and value equaling the offshore wind sector in 1991 were carefully assessed.

### Starting values and years for wave and tidal

Between 2010 and 2021, 30.2 MW of tidal stream energy converters have been installed in Europe since 2010, of which 11.5 MW were in the water in 2021 and 12.7 MW of wave energy converters have been installed, of which 1.4 MW were in the water in 2021 ^31^. A database of all those deployments was used to define the starting year and value.

First, the status of the technology developers in the database was checked and companies who have since disappeared or are hibernating for a prolonged time-period were filtered out. Second, the starting dates of entering the pre-commercial phase for both the wave and tidal sector were established based on publicly available data. The basic characteristics of the pre-commercial phase are: existence of Power Purchase Agreements (PPAs), first array installations, increased investor interest and policy support. Third, the starting year was matched with the cumulative capacity of still operating technology developers in that year resulting in the starting value.

For the tidal sector, 2016 was established as the year where the sector entered a pre-commercial phase. The dominating contribution came from Nova Innovation, Tocardo and the SIMEC Atlantis project (including turbines from Andritz Hydro). All three technology developers started to install the first turbines of larger-scale projects at that time. Together they had 3.05 MW installed which was considered the starting value for tidal energy in 2016. Additional active European technology developers who were not considered because they were in 2016 still more in a demonstration phase or deployed outside of Europe include Schottel Hydro, SME, Sabella, and ORPC.

For the wave sector, 2020 was established as the year where the sector entered a pre-commercial phase. In 2020 the constructions of the first MW scale systems started. Some prominent examples are the Corpower Ocean Wave Energy Converters array in Portugal, the Wello system in Spain and the full-scale demonstration of the Irish developer Ocean Energy in Hawaii. Others like EcoWavePower, Seabased and AW Energy demonstrated large-scale devices or arrays with PPAs and showed a large project pipeline. Combined with several additional offshore tested technology developers a critical mass of wave energy companies has been reached. Altogether this means that the entrance into a pre-commercial stage started in 2020. Altogether those companies had a cumulative capacity of 6.11 MW in 2020, which was chosen as the wave model starting value ( Table 1).

**Table 1.** Tidal and wave developers taken into consideration to choose the starting values.

Tidal developers	Wave developers
Tocardo (NL)	40South Energy (IT)
Nova Innovation (UK)	AMOG (AU)
SIMEC Atlantis Energy (UK)	AW-Energy (FI)
	CorPower Ocean (SE)
	Crestwing (DK)
	Eco Wave Power (SE)
	Floating Power Plant (DK)
	Fred Olsen Ltd (UK)
	GEPS Techno (FR)
	Hace (FR)
	Havkraft AS (NO)
	Marine Power Systems (UK)
	Ocean Energy (IE)
	Resen Energy (DK)
	Seabased (IE)
	SINN Power (DE)
	Voith Hydro (DE)
	Wave for Energy (IT)
	Wavenergy (IT)
	Wavepiston (DK)
	Waves4Power (SW)
	Wedge (SP)
	Wello Oy (FI)

An important difference between offshore wind and some bottom-fixed tidal systems on the one side and wave energy devices on the other side is the ease by which the latter can be deployed and towed back to shore for maintenance or improvements. Therefore, the cumulative capacity of companies still actively operating in the sector was used as the starting value for the wave energy sector, while only pre-commercial deployments were used for the tidal energy sector.

### Growth curve model

The first model - named "Growth curve model" - was developed based on an exponential fitting of the offshore wind cumulative capacity over the years. Firstly, the data was collected and only offshore wind farms commissioned and decommissioned between 1991 and 2021 in Europe with bottom-fixed foundations were taken into consideration. Secondly, the offshore wind turbine capacities were summed for each year and the decommissioned offshore wind turbines were subtracted whenever they have been decommissioned. Thirdly, a cumulative sum over the years was computed.

In order to apply the growth curve from the offshore wind sector to the marine energy sector curve fitting was applied. The curve fitting was performed to achieve the highest correlation between the function and the real curve, determined by the coefficient of determination *R* ^2^ values. The most appropriate function identified was an exponential split into three ten-year intervals (coefficients of determination between 0.915 and 0.993): (1) the "lag phase" from 1991 until 2001, (2) the "kick-off phase" from 2001 until 2011 and (3) the "growth phase" from 2011 until 2021 (and still going). If no intervals were considered an even higher average coefficient of determination was achieved (0.988). This, however, showed weak correlation with the datapoints in early years with low cumulative capacity. While the small values only led to small total derivation, the difference to what was really deployed was a factor 10 in the first 10 year interval (namely the "lag phase"). While this divergence did not weigh strongly for the overall correlation calculation it had a too strong influence on the prediction of the early growth of the sector.

The exponential coefficients of the growth curve were obtained by using the *curve_fit* Python function ( last access: July 2024), applied to the following equation:

$$\;P=a*e^{b*t_{y}}+P_{y,0}-a*e^{b*t_{y,0}}\;(1)$$

where *a* and *b* are the two coefficients computed by the *curve_fit* function, *P* the cumulative capacity in MW, *t _y_* the number of years since the year of reference, *P*_*y*,0_ and *t*_*y*,0_ the first values of the interval.

The coefficients of the three exponential functions were then applied to the wave and tidal energy sector. Depending on the starting point, a 30-year period was not always sufficient to reach 2050. In that case, the remaining years were extrapolated with the exponential coefficients of the last interval.

The coefficient of determination *R* ^2^ was computed using the following equation:

$$R^{2}=1-\frac{\sum_{i}{\left(y_{dataset,i}-y_{fitting,i}\right)}^{2}}{\sum_{i}{\left(y_{dataset,i}-{\bar{y}}_{dataset}\right)}^{2}}\;(2)$$

where *y*_*dataset*, *i*_ is the offshore wind cumulative capacity (from the dataset), ${\bar{y}}_{dataset}$ is the mean of the offshore wind cumulative capacities for each interval and *y*_*fitting*, *i*_ is the offshore wind cumulative capacity given by the model. The closer *R* ^2^ approaches 1 the better the correlation between the dataset and the applied function. *R* ^2^ values above 0.9 are acceptable.

### Doubling time model

The second model was based on the number of doubling events during the 30-year period. A doubling event is defined as the doubling of the installed capacity of the renewable energy technology globally or in a certain area. The doubling event computation is frequently used when analysing the behaviour of a sector. In the renewable energy sector, it is typically used for learning rates but it can also be useful for other techno-economic calculations.

The doubling time method is equivalent to the CAGR method which is used in other studies forecasting the growth of the marine energy sector ^18^.

Not only the number of doublings were computed but also the length of time between two doublings. These values gave the opportunity to have a more relatable idea of how a sector is growing. It was computed using the following equation:

$$\;T_{d}=(t_{2}-t_{1})*\frac{ln2}{ln\frac{P_{2}}{P_{1}}}\;(3)$$

where, *T _d_* is the length of time between two doublings, *t*_1_ the first year of the interval, *t*_2_ the last year of the interval, *P*_1_ the cumulative capacity for the first year of the interval in MW and *P*_2_ the cumulative capacity for the last year of the interval in MW.

The 30-year period was again split into six five-year intervals to have a high coefficient of determination ( *R* ^2^ = 0.995). A five-year interval was chosen and the growth rate inside the interval was assumed constant. The values used to compute the number of doublings were the same as for the growth curve scenario, i.e. the cumulative capacity over the years of the European bottom-fixed offshore wind farms, minus the decommissioned offshore wind turbines.

The number of doublings were computed using the following equation:

$$\;N=\frac{ln\frac{P_{2}}{P_{1}}}{ln2}\;(4)$$

where *N* is the number of doublings, *P*_1_ the cumulative capacity for the first year of the interval in MW, *P*_2_ the cumulative capacity for the last year of the interval in MW.

The number of doublings from the offshore wind sector was applied to the marine energy sector using the following equation:

$$\;P=P_{0}*2^{N}\;(5)$$

where *P* is the new value of the cumulative capacity for the last year of the interval and *P*_0_ the cumulative capacity value for the first year of the interval.

Between the two boundaries of the 5-year interval, the growth rate was assumed constant and the curve followed an exponential growth following the equation displayed below:

$$\;P(t)=P_{0}*2^{\frac{t}{T_{D}}}=P_{0}*e^{r*t}\;(6)$$

where *P*( *t*) is the cumulative capacity after a timeinterval t, *P*_0_ is the cumulative capacity for the first year of the interval, *T _d_* the length of time between two doublings (known thanks to the offshore wind sector computing), *r* the constant growth rate and *t* the length of time in years.

Similar to the growth curve model and depending on the starting point, a 30-year period was not always enough to reach 2050. In that case, the remaining years were extrapolated with the average of the number of doublings for the last three intervals known.

**Figure 2.**
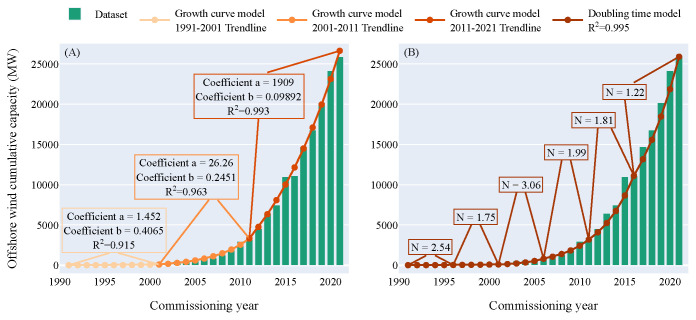
Offshore wind cumulative capacity over the commissioning years. ( **A**) Growth curve model. Coefficients a and b are the two coefficients computed by the curve_fit function used to draw the exponential fitting curve. The fitting curve is split into three ten-year intervals: the "lag phase", the "kick-off phase" and the "growth phase". The coefficient of determination is higher than 0.9 for the three intervals. - ( **B**) Doubling time model. N is the number of doublings. The whole period is split into six five-year intervals in order to follow the original dataset closely. The global value of the coefficient of determination is higher than 0.99 which indicates that the model is close to the original dataset.

The real offshore wind cumulative capacity and the trendlines from both models are displayed in Figure 2. The coefficients of the trendlines and the number of doublings displayed within the Figure were then used to compute the forecasts for the marine energy sector.

The coefficients of determination *R* ^2^ for the exponential fittings of the offshore wind cumulative capacity in the growth curve model and the doubling time model were all above 0.9 (and even higher than 0.95 for two intervals of the growth curve fitting and the doubling time fitting) which means that both models were close to the original dataset.

### Levelized Cost of Energy forecast

The growth- and doubling time models for wave- and tidal energy are combined with an projected LCoE forecast of Ocean Energy Europe (OEE) ^18^ based on an Ocean Renewable Energy (ORE) Catapult analysis ^19^. This forecast predicts the reduction of LCoE for wave- and tidal energy as the installed capacity increases ( Table 2). The tidal stream forecast follows the methodology described for the offshore wind sector ^19^, where the starting LCoE was determined to be 361 EUR/MWh for the 10 MW installed at the time ^18^. The wave forecast employed the same methodology ^19^, but uses the tidal stream’s learning rates, cost of capital and project development costs as well as a higher starting value as initial wave deployments were more expensive than tidal stream deployments at the time ^18^. A comprehensive description of the methodology adopted for the LCoE forecast is given in 18. A learning rate curve fit has been applied to Table 2, using the curve fit function of Python’s Scipy package ( last access: July 2024) to create a set of equations that provides an interpolated approximation of the per-capacity LCoE reduction for tidal ( Equation 7) and wave ( Equation 8).

**Table 2.** LCoE forecast. Columns 2 & 3 are adapted from 18, columns 4 & 5 are the calculated Learning Rates (LR).

Cumulative capacity (MW)	Tidal LCoE (€/MWh)	Wave LCoE (€/MWh)	Tidal LR (%)	Wave LR (%)
1	616	702	0.0	0.0
5	489	546	9.5	10.5
10	361	387	26.2	28.7
20	267	269	26.0	30.5
50	214	207	15.4	18.0
100	181	168	15.4	18.8
200	154	136	14.9	19.0
500	126	110	14.1	14.8
1,000	108	92	14.3	16.4
2,000	94	81	13.0	12.0

$$\;LCoE_{tidal}=736.37\cdot x^{-0.30}\;(7)$$

$$\;LCoE_{wave}=641.58\cdot x^{-0.26}\;(8)$$

Where, LCoE is the predicted LCoE [EUR/MWh] and x is the installed capacity of either wave- or tidal energy.

Equation 7 and Equation 8 have been applied to the installed capacity output by the growth- and doubling time models to obtain an approximation of the per-capacity LCoE.

### Scenarios

The main scenario presents the output of the growth curve model and the doubling time model based on the fits described above and using the identified starting years. Three scenarios with different starting years (2015, 2020, and 2025) and three scenarios with different starting cumulative capacities (1 MW, 5 MW, and 10 MW) are considered to determine the sensitivity of both models to varying parameters. Moreover, a best-case scenario is presented. Here, the "lag phase" fit observed for offshore wind is assumed to be avoided for the growth of ocean energy due to uncompromising policy support.

## Results and analysis

**Figure 3.**
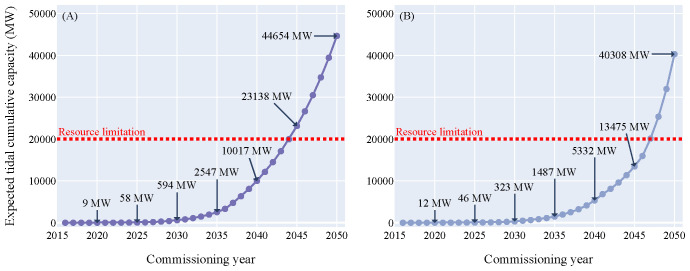
Expected tidal cumulative capacity over the commissioning years. ( **A**) Growth curve model - ( **B**) Doubling time model. Both models returned values with the same order of magnitude: between 46 and 58 MW could be deployed in 2025, between 323 and 594 MW in 2030 and between 40.3 and 44.7 GW in 2050. The red line displayed on the figure represents the tidal energy technical potential limitation in Europe which is around 20 GW. This limitation will be reached between 2044 and 2047 according to the models.

**Figure 4.**
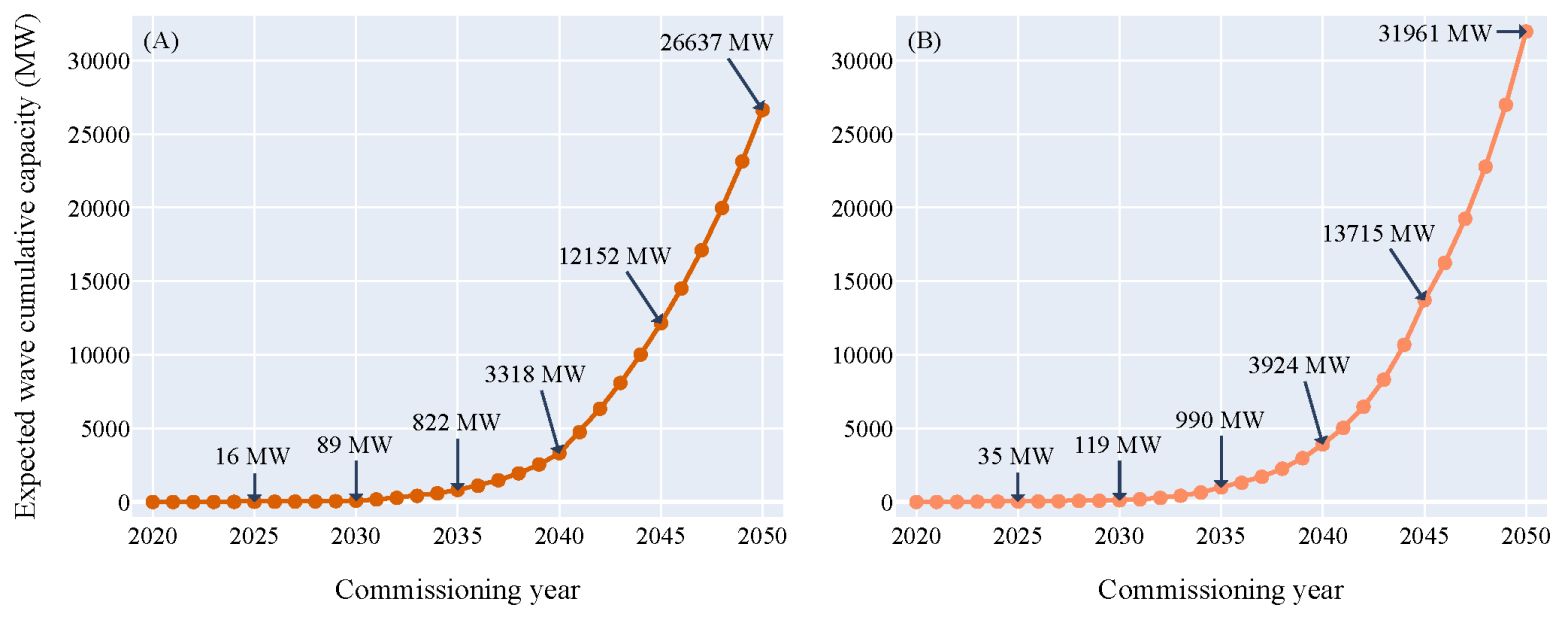
Expected wave cumulative capacity over the commissioning years. ( **A**) Growth curve model - ( **B**) Doubling time model. According to both models, between 16 and 35 MW of wave power could hit the water by 2025, between 89 and 119 MW by 2030 and between 26 and 32 GW by 2050. As the wave energy technical potential in Europe is not expected to be reached by 2050, the sector will likely keep growing significantly after 2050. The wave energy resource potential limitation (around 100 GW) is outside the graph boundaries and will not be met by 2050 according to both models.

In order to understand the growth of the marine energy sector in the next three decades the exponential growth and doubling time function were established for offshore wind ( Figure 2) and applied to the ocean energy sector. In 2016 and 2020 the starting capacity for tidal energy was estimated to 3.05 MW and the one of wave energy to 6.11 MW, respectively. Combining the starting capacities and years of wave and tidal with the exponential growth curve of offshore wind resulted in two growth curves per technology ( Figure 3, Figure 4).

The tidal energy sector entered its pre-commercial phase in 2016. Therefore, the amount of capacity that could be commissioned in the upcoming years according to the models is higher than for wave energy. The analysis showed that around 50 MW in 2025 (half of the European target) and between 320 and 600 MW in 2030 would be achievable. Between 2040 and 2044, 10 GW could be deployed and in 2050 between 40.3 and 44.7 GW of tidal power could theoretically be reached.

The wave energy sector reached its pre-commercial phase about four years after the tidal energy sector in 2020. Therefore, the deployed capacities in the upcoming years are lower than for tidal. In 2025, between 16 and 35 MW could be commissioned and between 89 and 119 MW in 2030. According to both models, 10 GW could be in the water in 2044. In 2050, between 26 and 32 GW of wave power could be deployed in Europe.

### Assessment of the resource potential

The results of the model were compared to the energy resource potentials to verify if the results are realistic. Depending on the technologies used to produce electricity, the efficiency varies. Therefore, there is a difference between the theoretical resource potential, which covers the total extractable amount of energy, and the technical resource potential, which includes limitations of current technologies.

For tidal energy, the technical resource potential limitation taken into consideration for this study is 20 GW ^19, 32^. The 20 GW limitation for tidal cumulative capacity could be reached in 2044 for the growth curve scenario and in 2047 for the doubling time scenario.

For wave energy, the technical energy potential in Europe is between 95 GW (Schlütter *et al.* ^33^), 286 GW ^34^ and as high as 50 TW ^35^. According to both models, this limitation is not expected to be reached by 2050. Therefore, the expected cumulative capacity for tidal and wave energy combined could reach between 46.6 and 52 GW in 2050.

### European targets

According to the models, wave and tidal capacity will be crucial to meet the 100 MW target by 2025 set by the European Union. The remaining capacity (less than 30 MW) could be partly filled with salinity gradient power plants, OTEC power plants or floating solar modules.

**Figure 5.**
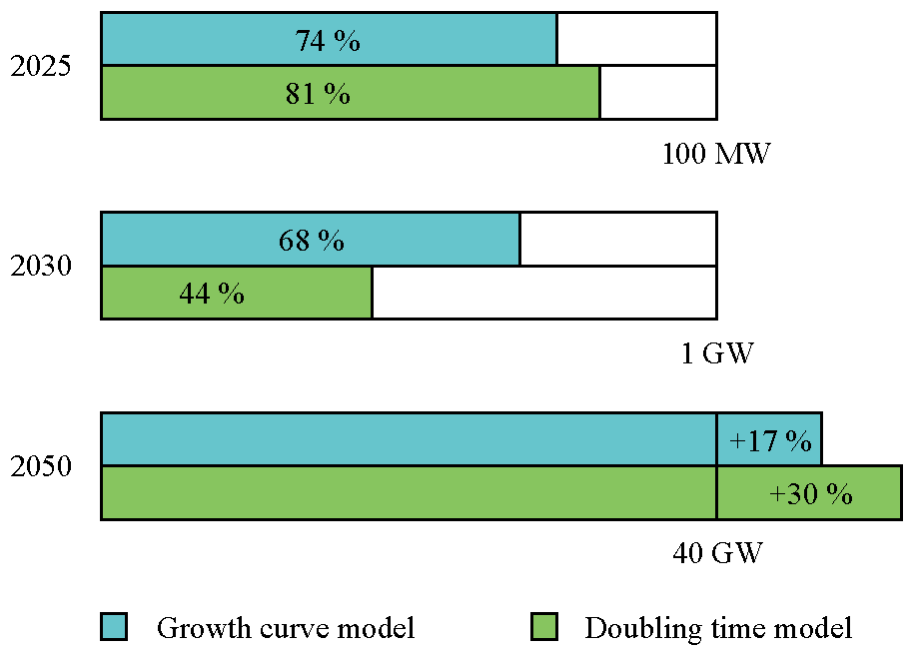
Completion percentage of the European targets. According to both models, the 2025 and 2030 targets will not be reached. Yet, the 2050 target could be overtaken. In order to get closer to the 2025 and 2030 targets, intensive support from European coastal countries are needed.

In the same way, the 2030 target of 1 GW will not be reached. Depending on the model considered, between 44 % and 68 % of the target could be filled with wave and tidal energy ( Figure 5). The remaining capacities would need to come from other marine energy technologies.

According to both models, the 40 GW target by 2050 is expected to be overtaken by 6 to 12 GW. Furthermore, adding floating solar, salinity gradient and OTEC capacities, the 2050 target can be even further overtaken. Important to note is that the UK was considered to contribute to the European targets.

### Levelized Cost of Energy forecast

**Figure 6.**
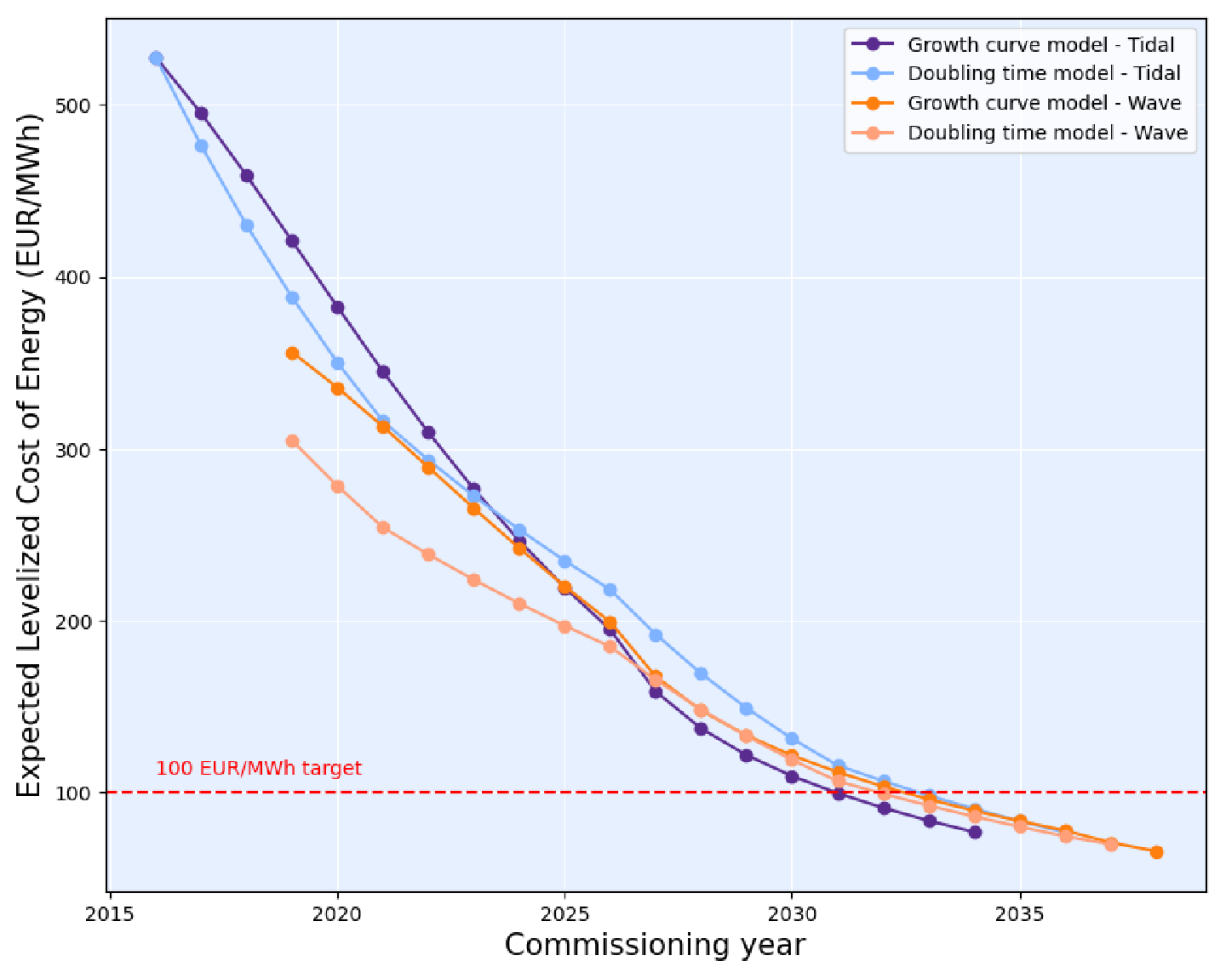
Expected LCoE over the commissioning years (the OEE dataset give the LCoE between 1 MW and 2000 MW of cumulative capacity). The 100 EUR/MWh target set by the European Commission could be reached, for both technologies, around 2035. The wave LCoE is higher than the tidal LCoE for the first capacities deployed but as more capacities hit the water, the LCoE for both technologies comes closer together.

Following the methodology given above, the expected LCoE over the commissioning years for each model and each technology are computed ( Figure 6).

It is generally accepted that a five-year delay between tidal LCoE and wave LCoE will occur ^15^. Yet, according to the models, the delay should be around five years during the first commercial deployment phase and then could shorten gradually until 2035 where the LCoE for both technologies will likely be equivalent.

The European Commission set targets for wave and tidal energy LCoE in the SET Plan ^15^: 100 EUR/MWh for tidal energy and 150 EUR/MWh for wave energy by 2030. The targets for 2030 will be slightly delayed. According to both models, the 100 EUR/MWh target for tidal energy could be reached between 2033 and 2035 and the 150 EUR/MWh target for wave energy could be met in 2031. The forecasts given by OEE and in this study show consistency with the newest developments in tidal energy. In particular the award of 41 MW of tidal energy in the UK at a strike price of 208 EUR/MWH aligns well with the predicted 214 EUR/MWh for 50 MW ^36^.

### Sensitivity analysis of both models

**Figure 7.**
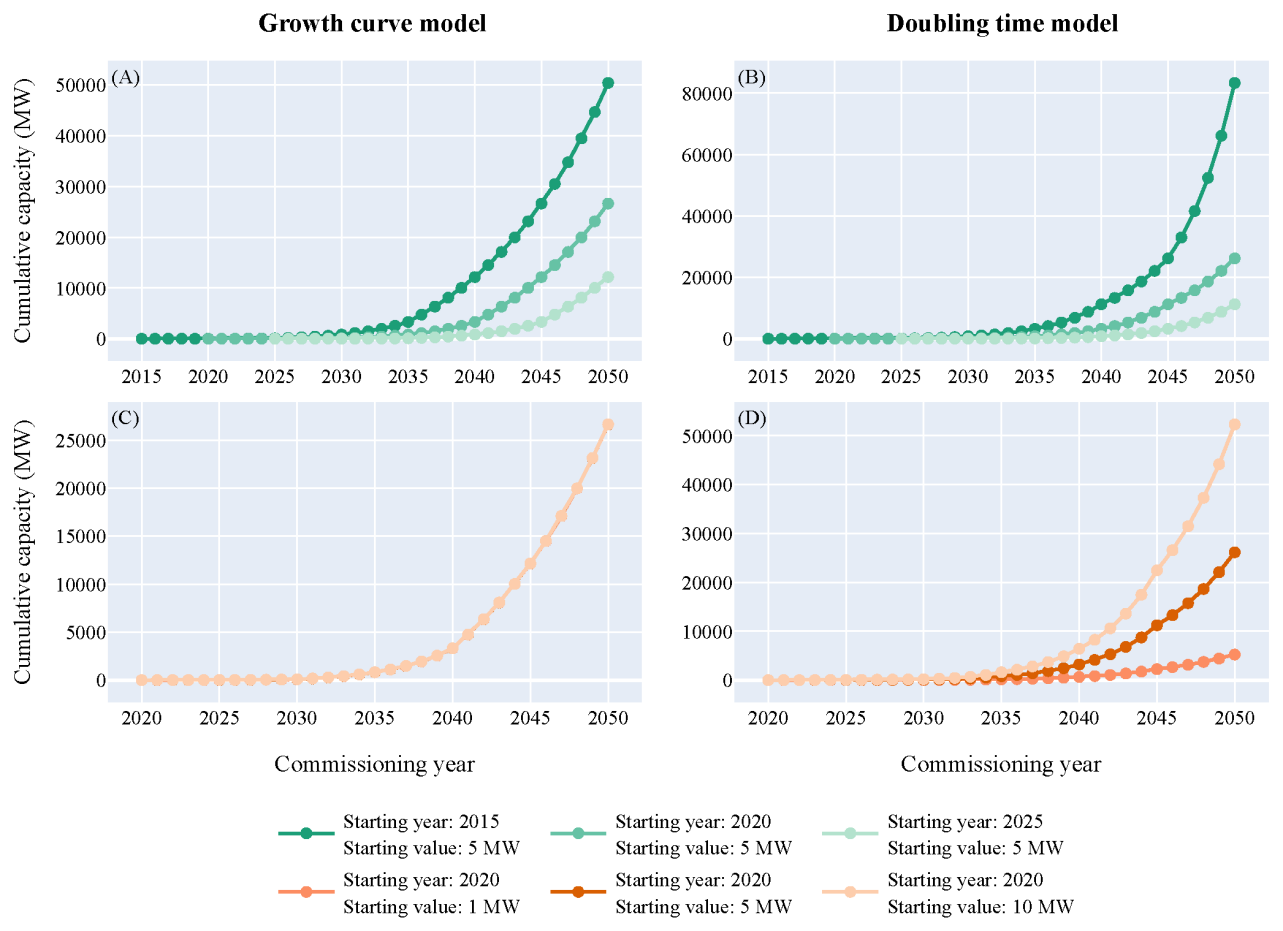
Sensitivity analysis of both models. ( **A**) Growth curve model, varying parameter=starting year - ( **B**) Doubling time model, varying parameter=starting year - ( **C**) Growth curve model, varying parameter=starting value - ( **D**) Doubling time model, varying parameter=starting value.

A sensitivity analysis was conducted for both models to understand how the models react when the starting year and the starting value vary ( Figure 7). Three different starting values (1 MW, 5 MW and 10 MW) and three different starting years (2015, 2020, 2025) were computed.

The variation of the starting year moves the curve to the left or to the right for both models. Therefore, as the growth is exponential, the expected capacity in 2050 changes drastically. For the growth curve model, if the starting year happens five years before, then the operating capacity in 2050 would be multiplied by 2.2. On the contrary, if the starting year happens five years later, then the operating capacity would almost be reduced by half. Regarding the doubling time model, the variation is even higher. A decrease of five years in the starting year leads to a 3.2 times higher capacity and an increase of five years leads to a 2.3 times lower capacity. The variation of the starting value does not have a significant effect for the growth curve model as it is only moving the curve up or down by the difference of the starting capacity. Therefore, a 5 MW variation of the starting value leads to a 5 MW difference in 2050, which is insignificant compared to the GW scale at this point. For the doubling time model, the variation of the starting value has a significant effect. If the starting capacity is doubled, then in 2050 the expected capacity will be doubled too. The starting year and the starting value are intrinsically linked but this analysis clearly highlights that a few years delay in the start of the first commercial deployments will significantly influence the operating capacities in 2050.

### Offshore wind support schemes

**Figure 8.**
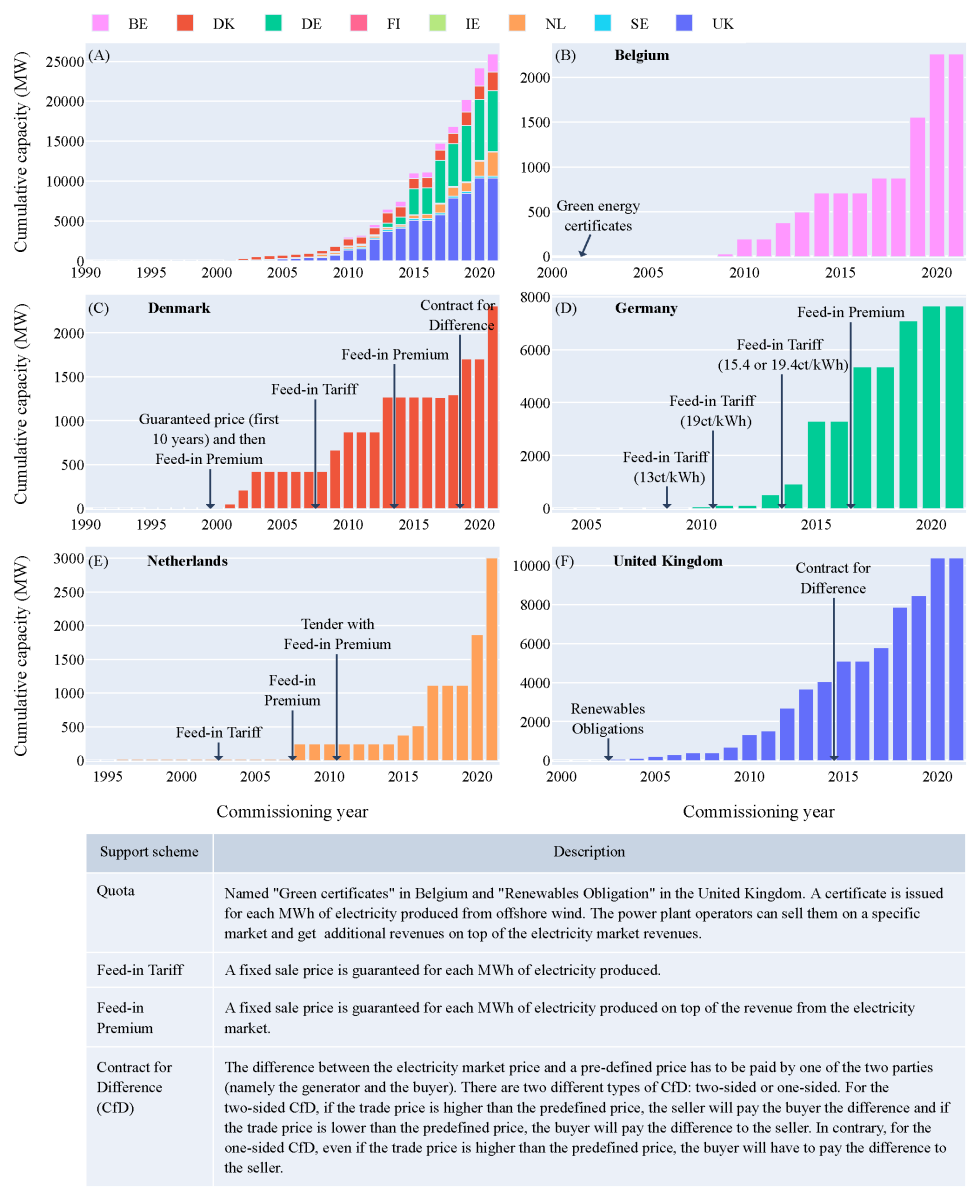
( **A**) Offshore wind cumulative capacity over the years for each European country - ( **B**) Offshore wind support schemes in Belgium - ( **C**) Offshore wind support schemes in Denmark - ( **D**) Offshore wind support schemes in Germany - ( **E**) Offshore wind support schemes in the Netherlands - ( **F**) Offshore wind support schemes in the United Kingdom. The first figure highlights the five European leading countries for offshore wind in terms of capacity deployed. Four different support schemes were used during the past 20 years: Feed-in Tariff, Feed-in Premium, Contract for Difference and Quota.

In 2022, five countries lead the European offshore wind market: Belgium, Denmark, Germany, the Netherlands and the United Kingdom with various offshore wind support mechanisms ( Figure 8). The average length of time between the awarding of an offshore wind farm and its commissioning is around five years ^37^. Therefore, the effect on capacity installed of new support schemes only materializes five years after. To reach the European targets for marine energy but also for offshore wind, shorter commissioning times are required. This result is consistent with the 5-year delay between the establishment of a new support scheme and the commissioning of offshore wind farms in various European countries ( Figure 8). Therefore the marine energy sector does not only need subsidies but also needs to reduce the time needed for commissioning of new offshore farms. The latter can be accomplished by an already established offshore supply chain, usage of satellite data to shorten offshore measurement campaigns, a detailed generally agreed on marine spatial plan and parallel permitting procedures instead of cascading ones taking all offshore renewables into consideration.

Similar to offshore wind, these subsidies will allow wave and tidal energy to mature and learn until they become profitable without direct subsidies from the government (e.g. Dutch offshore wind sector 2021). One way of structuring those different support schemes and subsidies in a transparent way ensuring the timely execution of projects, are offshore renewable tenders. Those tenders can specify what exactly the government supplies, what the boundaries are and what the goal of the tender is. A bidding system on those tenders increases competitiveness and value for money. An important indirect subsidy often interlinked with a tender structure is the provision of the offshore grid. This comes with the positive side-effect that the offshore grid will not only be optimized on the project developers profit, but also on energy security and overall system costs.

### Best case scenario

If an uncompromising policy support is set quickly to assist the tidal and wave energy sector growth, a very fast development of new or hibernating projects can be expected. It could also lead to a consolidation of technologies with a standardization of components used in wave and tidal devices. This standardization will further reduce the costs as supply chains become more competitive and reliable. Moreover, the tidal and wave sectors could benefit from the existing offshore supply chain developed for the offshore wind sector.

By introducing support schemes to support the sector immediately the "lag phase" that occurred for the offshore wind sector in the 1990s is avoided and the 2025 and 2030 European targets can be met as well.

**Figure 9.**
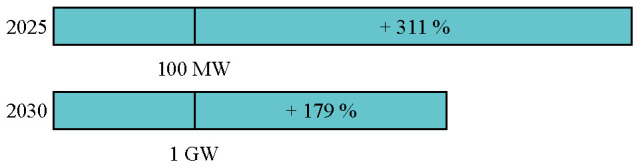
Completion percentage of the European targets in the best case scenario using the growth curve model. The European targets could be greatly overtaken if actions are taken now.

**Figure 10.**
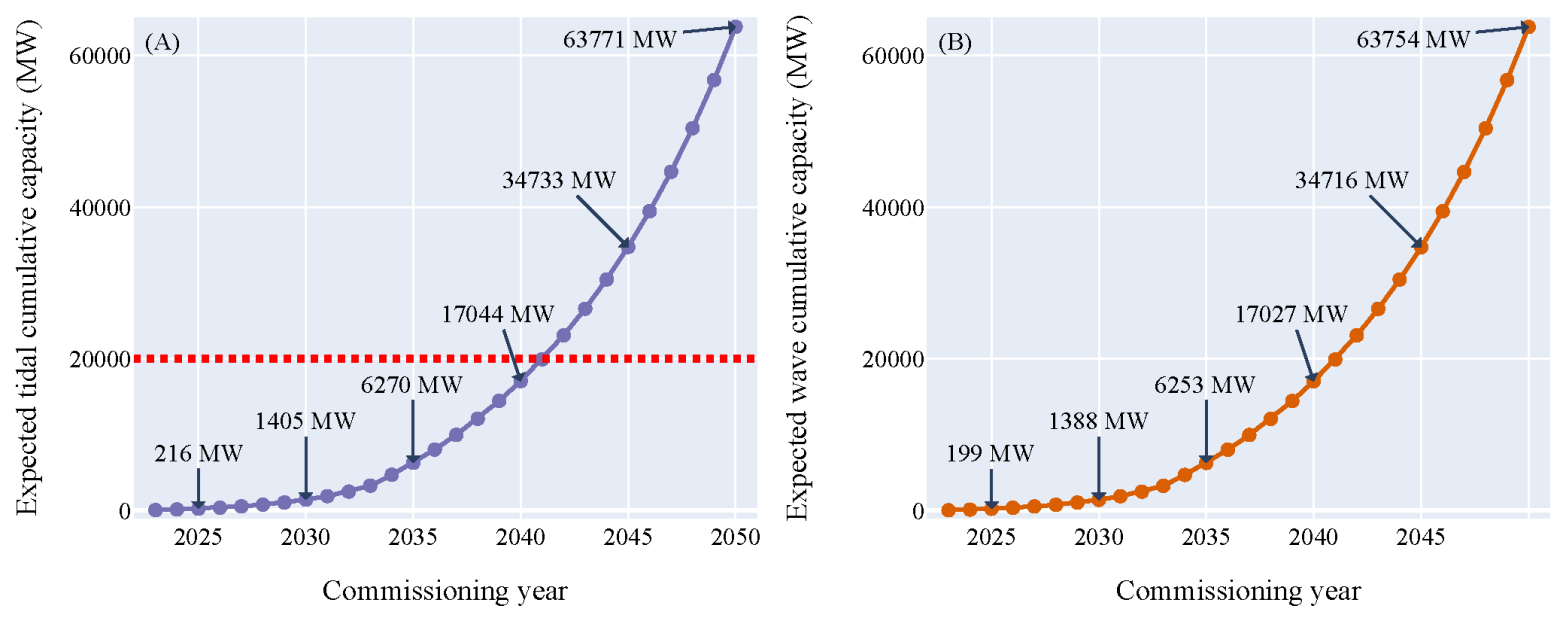
Expected tidal and wave cumulative capacities according to the best case scenario using the growth curve model. ( **A**) Tidal - ( **B**) Wave Page

The second phase of the offshore wind development started around 2001 with the introduction of support schemes. When computing the growth curve model using 2001 as the new starting year for offshore wind and 2023 as the starting year for both the tidal energy sector and the wave energy sector (see Appendix, Figure 10), the 2025 and 2030 European targets regarding the operating capacity could be greatly overtaken ( Figure 9). The starting values taken into consideration are 26.58 MW for the tidal sector and 9.57 MW for the wave sector. These values are obtained by computing the growth curve model using the initial starting points and considering the expected cumulative capacity in 2023.

## Discussion and conclusion

A huge amount of untapped energy is located in our oceans and seas. Ocean energy technologies like wave and tidal are needed to tap this last big reservoir of renewable energy and are needed to meet a global "Net Zero Emission" energy system. Different pathways can reach this target, where a highly renewable energy system is likely to be the most efficient, also cost-wise ^38^. Although ocean energy has been considered in small-scale 100% renewable energy systems on islands ^39^, for global 100% renewable energy systems, wave, and tidal energy are rarely included as part of the renewable energy mixture ^38^. Europe is leading the ocean energy sector but the US and China are also catching up. Only by a fast implementation of strong support policies to further develop it, Europe will stay on top generating unique export opportunities. Doing so and considering all limitations both the wave and tidal sector will likely grow similarly to offshore wind. In the last three decades, more than 25 GW of offshore wind was commissioned in European waters. This study, based on the development of the offshore wind sector, forecasts around 50 GW of wave and tidal operating capacity in Europe by 2050. According to the models developed for this study, the full technical potential of 20 GW of tidal energy could be deployed between 2044 and 2047. Following the offshore wind growth, intermediate values between 46 and 58 MW in 2025 and between 323 and 594 MW in 2030 are realistically achievable. Regarding the wave operating capacity, between 26 and 32 GW could be deployed by 2050. As the resource potential in Europe for wave energy is far higher than that, further growth after 2050 is likely. Moreover, between 16 and 35 MW of wave energy could be deployed by 2025 and between 89 and 119 MW by 2030. According to the models and based on the limited policy commitments of European coastal countries to this date, the European targets set for 2025 and 2030 (respectively 100 MW and 1 GW of marine energy) will not be met. In total, between 74 and 81 MW of wave and tidal energy could be deployed by 2025 and between 442 and 683 by 2030. Other ocean energy technologies such as OTEC and salinity gradient will unlikely be enough to fill the gap in those years. In 2050, taking into consideration the tidal resource limitation, between 46.6 GW and 52 GW could be deployed, greatly overtaking the European target of 40 GW. Based on the results from both models and the LCoE forecasts from OEE, the 100 EUR/MWh LCoE target for tidal energy could be met in 2033, and the one for wave energy in 2035. This cost reduction makes the sector cost-competitive to other energy sources. However, considering the international developments in 2022 and the tremendously increased cost of electricity one could argue that those technologies are already price competitive if being installed in arrays. Thus, both tidal and wave can be considered, in terms of resource availability and economic competitiveness, as part of a future high renewable energy system and could provide a step forward towards a global "Net Zero Emission" energy system, as well as diversify the renewable energy mixture, enhancing energy security and grid reliability ^3, 40, 41^.

The main assumption of this study is that the tidal and wave energy sectors will behave the same way as the offshore wind sector did in the past. Wave and tidal could develop faster than offshore wind, as they can build upon challenges already overcome by offshore wind, such as grid connection, operation & maintenance, etc. However, if we want the marine energy sector to grow the same way, or even more faster than the offshore wind sector, we need to be sure that the marine energy sector benefits from the same or, given the time constraint at hand, even better support from European coastal countries. Moreover, the sooner the supportive measures will occur, the higher the operating capacity will be. As seen with the sensitivity analysis, a five-year delay can lead to almost a 50 % decrease in the operating capacity in 2050.

The growth of the offshore wind sector should be taken as an example in terms of support schemes, but the marine energy sector can learn from previous misconceptions. In particular a reduction of delays between the establishment of a policy and when it is applied to a project and the increased speed of permitting and consenting will be critical. At the European level and for some European countries, the first positive developments are visible (European marine energy targets, European initiative to standardize and fasten permitting procedures, UK’s contract for difference on tidal energy, commitments of the Portuguese and Spanish government, etc.). Overall, the marine energy sector shows great potential to support the European and global climate targets.

## Data Availability

Zenodo. European offshore wind farms and marine energy deployments. DOI: 10.5281/zenodo.7938413. This project contains the following underlying data: Offshore_wind_farm_european_deployements.xlsx (European offshore wind farms dataset used to forecast the development of marine energy in Europe in the upcoming three decades). Tidal_Energy_Converters_European_deployements.xlsx (Tidal energy converter deployments in Europe dataset used to forecast the development of marine energy in Europe in the upcoming three decades). Wave_Energy_Converters_European_deployements.xlsx (Wave energy converter deployments in Europe dataset used to forecast the development of marine energy in Europe in the upcoming three decades) Data are available under the terms of the Creative Commons Attribution 4.0 International license (CC-BY 4.0).
