# Supplementary material for: Mapping out the scenarios of ocean energy scale-up based on the development of offshore wind
Source: Open Res Eur. 2025 Jan 28;3:102. Originally published 2023 Jun 20. [Version 2] doi: 10.12688/openreseurope.15906.2 (PMC11993849; doi:10.12688/openreseurope.15906.2)
Supplement: Peer review history

# Reviewer report: Reviewer response for version 2

**DOI:** 10.21956/openreseurope.19897.r50412

**Published:** 2025-04-12

**Authors:** Christian Breyer

**Affiliations:** 1 School of Energy Systems, LUT University, Lappeenranta, Finland

Thanks for the revised manuscript. The very comprehensive revisions made a substantially improved manuscript. 

 Only one new comment remains: p. 9, 3 ^rd^ paragraph: the potential for Europe according to Satymov et al. (2024) is 4.5 TW (see Supplementary 2, tab 2050, cell Y6), not the used 50 TW. With that single number polishing all should be fine.

Is the study design appropriate and does the work have academic merit?

Yes

Is the work clearly and accurately presented and does it cite the current literature?

Partly

If applicable, is the statistical analysis and its interpretation appropriate?

Yes

Are all the source data underlying the results available to ensure full reproducibility?

Yes

Are the conclusions drawn adequately supported by the results?

Yes

Are sufficient details of methods and analysis provided to allow replication by others?

Partly

Reviewer Expertise:

renewable energy, energy systems, energy transition, ocean energy

I confirm that I have read this submission and believe that I have an appropriate level of expertise to confirm that it is of an acceptable scientific standard.

# Reviewer report: Reviewer response for version 2

**DOI:** 10.21956/openreseurope.19897.r50919

**Published:** 2025-03-04

**Authors:** Alain Ulazia Manterola

**Affiliations:** 1 University of the Basque Country (UPV/EHU), Gipuzkoa-Eibar, Spain

This work studies LCOE of marine energies (wave and tidal) in analogy to offshore wind, to project it to future decades according to the learning curve presented by wind turbines in the past.

 Every analogy can be dangerous, and the possible weakness of this analogy should be also analized, within the limitations of the work.

 The presented python code for fitting the curve should be improved the consider confidence intervals of the prediction. Similarly, a relative error should be assigned to the prediction.

 Not only the fitting process, the input data also show an intrinsic uncertainty and this should be considered in the final prediction range.

Is the study design appropriate and does the work have academic merit?

Partly

Is the work clearly and accurately presented and does it cite the current literature?

Yes

If applicable, is the statistical analysis and its interpretation appropriate?

Partly

Are all the source data underlying the results available to ensure full reproducibility?

Yes

Are the conclusions drawn adequately supported by the results?

Yes

Are sufficient details of methods and analysis provided to allow replication by others?

Partly

Reviewer Expertise:

resource assessment for marine energies, data science, climate models, reanalysis

I confirm that I have read this submission and believe that I have an appropriate level of expertise to confirm that it is of an acceptable scientific standard, however I have significant reservations, as outlined above.

# Reviewer report: Reviewer response for version 2

**DOI:** 10.21956/openreseurope.19897.r50470

**Published:** 2025-02-19

**Authors:** Damon Howe

**Affiliations:** 1 University of Tasmania, Sandy Bay, Australia

The manuscript presents a methodology and analysis for the potential contributions that wave and tidal energy can make in the European renewable energy mix given similar growth and development trends to fixed-bottom offshore wind. 

 General comments:

 The manuscript could do with a final proof-read for grammar and clarity as there were some instances where sentence structure did not help to support the arguments being made. However, overall the manuscript followed a logical flow.

 Further details need to be provided around the application of the offshore wind trends to the wave and tidal predictions. There is a lack of discussion around the limitations of the modelling. 

 Introduction:

 - Depending on the target audience of the manuscript, it might be worthwhile to define (in simplistic terms) what the metric of LCOE measures. 

 - A question must be asked regarding the use of the capacity growth related to bottom-fixed offshore wind being applied to tidal, and particularly wave in order to provide sufficient data. While these ORE sectors do share many similarities, as mentioned, one major distinction which differentiates the sectors, and has subsequent impact on metrics such as learning rates, is the lack of technological convergence for wave energy (tidal to a lesser degree). Offshore wind is dominated by HAWTs, where wave energy has yet to converge (which also raises the question, is a one-size-fits-all approach best?). I am wondering whether this complexity has been considered in the modelling? If so, what assumptions have been made?

 Methods:

 - Was the exponential growth curve derived from fixed offshore wind directly transferred to wave and tidal? Or were the coefficents in Equation 1 individually derived for each respective ORE sector? 

 - In Equation 6, was the growth rate, r, defined as that of the offshore wind, and then subsequently applied to wave and tidal? This is still somewhat unclear in the methodology description, and would relate to the previous points made. 

 - A section should be included to address any limitations/simplifications/assumptions present in the two models. 

 Discussion and Conclusions:

 - More details required about main assumption, how likely is this given that offshore wind is now dominating the market?

Is the study design appropriate and does the work have academic merit?

Yes

Is the work clearly and accurately presented and does it cite the current literature?

Partly

If applicable, is the statistical analysis and its interpretation appropriate?

Yes

Are all the source data underlying the results available to ensure full reproducibility?

Yes

Are the conclusions drawn adequately supported by the results?

Partly

Are sufficient details of methods and analysis provided to allow replication by others?

Partly

Reviewer Expertise:

Offshore renewable energy, particularly wave energy.

I confirm that I have read this submission and believe that I have an appropriate level of expertise to confirm that it is of an acceptable scientific standard, however I have significant reservations, as outlined above.

# Reviewer report: Reviewer response for version 2

**DOI:** 10.21956/openreseurope.19897.r50469

**Published:** 2025-02-18

**Authors:** Abel Arredondo Galeana

**Affiliations:** 1 University of Strathclyde, Glasgow, UK

This paper addresses the very relevant topic of forecasting the cumulative capacity and reduction in LCOE of marine renewables towards 2050. The paper uses the growth profile of bottom fixed offshore wind energy to derive cumulative capacity growth coefficients to be applied to tidal and wave energy. Then, the LCOE is predicted based on the predicted growth of the tidal and wave energy sector.

 Naturally, the approach considered in this work could have certain limitations. For example, the fact that wave energy has not converged to one type of device, as offshore wind did, or the fact that wave energy addresses floating technology and not bottom fixed. Tidal energy, although more similar to offshore wind in terms of device, operates in a submerged manner, and in general, close to seabed or near the water surface. Furthermore, different political, warfare, climate or other type of global event, can affect the growth patterns observed in previous decades. Hence, these limitations should be mentioned in the discussions of the paper.

 Nonetheless, novel and creative approaches are needed to predict the growth and LCOE reduction of tidal and wave energy, because growth and cost predictive models are of interest to all the relevant stakeholders to meet net zero targets.  Furthermore, the paper addressed already two high quality peer reviews in the previous round. Therefore, I recommend the paper for indexing subject to the following minor reviews:

 Introduction:

- Fix typo: salinity grandient > salinity gradient
- “positive policy developments in the ranging from the innovation fund and Horizon Europe program *” > *remove “in the”
- “Only a few,” > missing verb >”Only a few have attempted to include them,…”
- “to have more energy system reports, including ocean energy in a reliable way,” > insert comma

Methods:

 Tidal energy:

- I would note here that most of the examples considered are for fixed bottom tidal turbines and examples of floating tidal turbines are not considered. Possibly discuss the case of Orbital Marine Power that have deployed 2 MW in 2021, as discussed in the reference: *D. R. Noble, K. Grattan, and H. Jeffrey, ‘Future economic potential of Tidal Stream & Wave Energy in Scotland’, The University of Edinburgh, Jan. 2025 for possible benchmark comparison cases.*
- Table 1 seems very asymmetric in terms of tidal developers. Although only these three developers were considered, I would include a few more developers here. See for example Table 1 of [Ref 1].

Wave energy:

- For the wave sector – 2020 was established as the year were the sector entered a precommercial scale. > I suggest making a note here to mention that wave energy was pioneered in the 1970s, and has been around even before tidal turbines. However, policy changes and the simultaneous growth of wind power delayed the sector, and arguably, the lag-phase for wave energy could be much longer than what this current paper predicts.
- Maybe worth motioning here the case of Mocean energy, which has been successfully supported by Wave Energy Scotland to carry out a large-scale demonstrator.

Additional notes:

- Sensitivity analysis - Figure 7c seems to have only one curve as opposed to Figure 7a, 7b and 7d. Please check.
- Figure 8 – Please define acronyms used for each country. For example, FI, IE, SE are not defined in the manuscript.

Discussion:

- “Following the offshore Following the offshore wind growth, intermediate values between 46 and 58 MW in 2025 and between 323 and 594 MW in 2030 are realistically achievable.” – Please clarify that this statement refers to tidal energy as observed in Fig. 3.
- Pg 13 - Please change “more faster” to “faster”

Is the study design appropriate and does the work have academic merit?

Yes

Is the work clearly and accurately presented and does it cite the current literature?

Yes

If applicable, is the statistical analysis and its interpretation appropriate?

Yes

Are all the source data underlying the results available to ensure full reproducibility?

Yes

Are the conclusions drawn adequately supported by the results?

Yes

Are sufficient details of methods and analysis provided to allow replication by others?

Yes

Reviewer Expertise:

Marine renewables; Tidal energy; Wave Energy; Offshore renewables

I confirm that I have read this submission and believe that I have an appropriate level of expertise to confirm that it is of an acceptable scientific standard, however I have significant reservations, as outlined above.

## References

[1] : Tidal current energy harvesting technologies: A review of current status and life cycle assessment. *Renewable and Sustainable Energy Reviews* .2023;179: 10.1016/j.rser.2023.113269 10.1016/j.rser.2023.113269

# Reviewer report: Reviewer response for version 1

**DOI:** 10.21956/openreseurope.17180.r33209

**Published:** 2023-10-24

**Authors:** Christian Breyer

**Affiliations:** 1 School of Energy Systems, LUT University, Lappeenranta, Finland

The manuscript presents an investigation of scenarios on how ocean energy can be scaled up in Europe using learnings from wind offshore.

**Main items:**

- How the capacity of tidal and wave is linked to LCoE (as shown in Tab. 2) remains unclear. This gap needs to be filled. This gap may be larger, as directly or at least indirectly the learning rate seems to be required, while this aspect is not addressed in the paper so far.
- It may be supportive to highlight the existing research gap in energy system studies that often ignore wave and tidal power, as this leads to a lower awareness of the existing options.
- More scientific literature in the field of research helps for providing more context.

In the following you find all comments in detail, while most are smaller items

**Abstract:**

 In the Results section of the Abstract you refer to targets of the European Commission and link then to European coastal countries. Are the ‘European coastal countries’ limited to the member states of European Union? There is a large potential in the UK, while the UK does not belong to EU-27, and thus, the UK cannot contribute to the targets of the EC. Wording clarification may be helpful.

**Keywords:**

 Are ‘wave power’ and ‘tidal power’ more clear to highlight the electricity harvesting aspect of the more general term of ‘wave’ and ‘tidal’?

**Introduction:**

 1 ^st^ paragraph: wave and tidal are the most advanced ocean energy technologies, next to offshore wind, better to expand the wording for inclusion of offshore wind. More literature references for the wave and tidal potential may be helpful. Gunn and Stock-Williams (2012 ^1^) find up to 18,500 TWh and Mork *et al*. find up to 26,100 TWh ( https://doi.org/10.1115/OMAE2010-20473). Nevertheless, the study is on Europe, thus, the potential for wave power and tidal power in Europe would be of particular interest. You may also mention the potential of offshore wind, so that the three technologies you focus on in the paper are all presented with their potential in Europe.

 2 ^nd^ paragraph: better to clarify the targets for ocean energy in addition to offshore wind, as the latter may have separate targets, given the numbers presented.

 4 ^th^ paragraph: it may be worth mentioning whether in other parts in the world, in particular in Asia, one can observe progress in the deployment of wave and tidal power. Experience shows that a major breakthrough in other world regions may induce strong development in other regions as well. Please check the 4 ^th^ sentence for the grammar.

 5 ^th^ paragraph: ‘forecast’ typically describes periods up to 5 not more than 10 years, while longer periods in the order of decades may be better described by ‘projection’ for reflecting a higher degree of uncertainty.

 It would be interesting to add one paragraph on literature on the role of wave and tidal power for Europe. Are the two technologies mentioned in WEO reports of the IEA for Europe? Is there scientific literature on the role of the two technologies, for Europe, or for single countries? For the IEA WEO I checked it but only global numbers are reported in recent reports, no numbers for Europe. On a country basis at least a recent study for the UK, which found up to 42 GW installed capacity in the UK and Ireland until 2050 (Alvarez-Silva *et al.*, 2016 ^3^), this may be valuable for this research as it documents a value add of wave power for the energy transition from an energy system perspective. In the energy [r]evolution scenario of Greenpeace one can find 53 GW of ocean energy installed in OECD Europe by 2050 ( https://www.greenpeace.org/static/planet4-canada-stateless/2018/06/Energy-Revolution-2015-Full.pdf; Tab. 13.4.18), which may be a valuable reference, given the low coverage of ocean energy in energy scenarios. More related research may be quite helpful for the aims of the paper.

**Methods:**

 There is an unexpected break in the flow from the 2 ^nd^ to the 3 ^rd^ paragraph (from wind to wave/tidal). Having the section headline expanded to ‘Starting values and years for wave and tidal’ may ease that a bit.

 p. 4, 1 ^st^ paragraph: ‘power purchase agreement’ is used again as a word, while the abbreviation PPA was introduced earlier in this section. Another example is CAGR what’s again used in the long version on the next page, while the abbreviation was introduced before. Please check for all abbreviations that they are used consequently, once the abbreviation was introduced.

 Table 1: Sweden is abbreviated with ‘SW’, while the typical convention should be ‘SE’, please check for the entire manuscript.

 p. 4 right column, 3 ^rd^ paragraph: ‘bad correlation’ may be better as 'poor' or 'weak' correlation.

 p. 5, 1 ^st^ paragraph: three exponential functions are mentioned. I may be helpful to mention the three phases so that it is explicitly clear to what ‘three’ refers.

 p. 5 (and others): please check all used abbreviations whether they are introduced. For instance OEE and ORE appears, while this should be abbreviations.

 p. 5/6: section on LCoE is important for the entire paper. Please introduce the formula for LCoE.

 p. 6 left column bottom: “… the values given in the table.” Which table? Please refer explicitly to the table referred to in the paper.

 p. 6: Tab. 2 is not referenced in the text, please embed it into the text. Where are the starting values for tidal and wave LCoE for 1 MW from? These are among the most important references, as the derived costs are directly linked to these starting values. Please discuss the validity of these starting values, as they have a very strong impact on the results. You may consider a sensitivity for these values. The link of LCoE to the capacity remains unclear. What formula links LCoE to the capacities? Is a learning rate applied? If so, which value(s) and based on what reference? The Tab. 2 seems to be central, while the impact of the capacity on the LCoE remains unclear.

**Results and analysis:**

 p. 8, 3 ^rd^ paragraph: it may be worth checking additional literature for the wave power potential of 95 GW in Europe. There is unpublished research with much higher values (more than an order of magnitude more, however, for entire Europe, thus, explicitly including the UK and Norway), therefore, other literature may also find higher values. Please also clarify for the wave power potential for Europe, whether entire Europe (incl. the UK, Norway, Iceland, etc.) is meant, or EU-27. For the case of ocean energy this makes a considerable difference. The best may be to clarify across the entire paper whether EU-27 or entire Europe is meant, whenever the term ‘Europe’ is used. In the right column it is mentioned that the UK is assumed to contribute to the EU targets. That most important assumption is better mentioned earlier in the paper, when the term ‘Europe’ is used for the first time in the context of this paper.

 p. 8, section European targets: please add per additionally mentioned category (salinity gradient, OTEC, floating PV) at least one scientific reference. Good literature may be (please check for yourself): Alvarez-Silva *et al.* (2016 ^3^); Langer *et al.* (2020 ^4^); Shi *et al.* (2023 ^5^); Breyer *et al.* (2023 ^6^); however, at least OTEC seems not to be that attractive for Europe given the tropical water temperature required, while the Mediterranean may develop towards that directly due to climate change, unfortunately.

 p. 8, Fig. 5: link to the (new) methods how the LCoE decline over time. What is the learning rate? How is the cost decline calculated? It’s not clear in the paper how this is calculated.

 p. 10, 1 ^st^ paragraph: ‘deployment’ may be better suited then ‘journey’.

 p. 10, section offshore wind: it may be helpful to better explain what ‘MetOcean’ shall be.

 p. 10, section ‘Best case scenario’. It may be helpful to introduce the ‘Best case scenario’ already in the Methods section, so that readers are prepared on different scenarios or respective variations. That may be done best in a paragraph on scenarios to be considered.

**Discussion and conclusion:**

 p. 10, 1 ^st^ paragraph of the section. The term ‘net zero emission’ links nicely to the same from the Introduction. It may be helpful to expand the section on ‘net zero emissions’ a bit as this is clearly set in the European Green Deal. Different pathways are possible to reach that aim, while highly renewable energy system transition options may be most likely, also cost-wise. This and a review on comparable studies was published here (Breyer *et al.*, 2022 ^7^), where wave and tidal power is missing in almost all studies, and that research gap may be documented with this overview, while the previously mentioned paper on the UK clearly indicates that latest research may be able to close this gap. In that regard, it may be helpful that at least for three European islands wave and tidal power systems had been investigated as part of a highly renewable energy system and published in scientific articles (Meschede *et al.*, 2022 ^8^; see Tab. 5), as detected for the cases of El Hierro, Faroe, and Sardinia. Not sure whether this may be a helpful indication of upcoming wave and tidal power systems for the benefit of the paper, as island energy systems.

 You may mention, that wave and tidal power could also develop faster than wind offshore, since many challenges (such as grid connection, O&M, harbour logistics, etc.) have been fixed for wind offshore, thus, wave and tidal power can build on that, which should enable a faster growth potential.

**References:**

 Please expand the references across the paper for more scientific references in scientific journals. The present list of references lists 16 references, thereof only one is from a scientific journal. Any share of 25%, better 33% up to 50% of peer-reviewed journal references should be a target to provide a broader context to the scientific literature in the field of renewables, the energy transition, in particular in Europe, ocean energy in general, but in particular for wave power and tidal power, and their link to offshore wind.

Is the study design appropriate and does the work have academic merit?

Yes

Is the work clearly and accurately presented and does it cite the current literature?

Partly

If applicable, is the statistical analysis and its interpretation appropriate?

Yes

Are all the source data underlying the results available to ensure full reproducibility?

Yes

Are the conclusions drawn adequately supported by the results?

Yes

Are sufficient details of methods and analysis provided to allow replication by others?

Partly

Reviewer Expertise:

renewable energy, energy systems, energy transition, ocean energy

I confirm that I have read this submission and believe that I have an appropriate level of expertise to confirm that it is of an acceptable scientific standard, however I have significant reservations, as outlined above.

## References

[1] : Quantifying the global wave power resource. *Renewable Energy* .2012;44: 10.1016/j.renene.2012.01.101 296-304 10.1016/j.renene.2012.01.101

[2] : Offshore versus onshore: The underestimated impact of onshore wind and solar photovoltaics for the energy transition of the British Isles. *IET Renewable Power Generation* .2023;17(13) : 10.1049/rpg2.12840 3240-3266 10.1049/rpg2.12840

[3] : Practical global salinity gradient energy potential. *Renewable and Sustainable Energy Reviews* .2016;60: 10.1016/j.rser.2016.03.021 1387-1395 10.1016/j.rser.2016.03.021

[4] : Recent progress in the economics of ocean thermal energy conversion: Critical review and research agenda. *Renewable and Sustainable Energy Reviews* .2020;130: 10.1016/j.rser.2020.109960 10.1016/j.rser.2020.109960

[5] : Review on the development of marine floating photovoltaic systems. *Ocean Engineering* .2023;286: 10.1016/j.oceaneng.2023.115560 10.1016/j.oceaneng.2023.115560

[6] : Role of Solar Photovoltaics for a Sustainable Energy System in Puerto Rico in the Context of the Entire Caribbean Featuring the Value of Offshore Floating Systems. *IEEE Journal of Photovoltaics* .2023; 10.1109/JPHOTOV.2023.3319022 1-7 10.1109/JPHOTOV.2023.3319022

[7] : Reflecting the energy transition from a European perspective and in the global context—Relevance of solar photovoltaics benchmarking two ambitious scenarios. *Progress in Photovoltaics: Research and Applications* .2022; 10.1002/pip.3659 10.1002/pip.3659

[8] : A review of 100% renewable energy scenarios on islands. *WIREs Energy and Environment* .2022;11(6) : 10.1002/wene.450 10.1002/wene.450

# Response

**Published:** 2024-08-08

**Authors:** Benjamin Lehner

Thank you Christian for this detailed and valuable feedback and excuses for the delayed reply. We will upload the latest version in the coming days but please find below already our answers to your remarks ( *in cursive)* one by one with the replies underlined.  CB.1.1: In the Results section of the Abstract you refer to targets of the European Commission and link then to European coastal countries. Are the ‘European coastal countries’ limited to the member states of European Union? There is a large potential in the UK, while the UK does not belong to EU-27, and thus, the UK cannot contribute to the targets of the EC. Wording clarification may be helpful.

BL 1.1: Thank you for the comment, we agree that the differentiation between Europe / EU-27 / EU-27 plus associated countries are not clear enough in the paper. For clarity we added the following subsentence (bold) in the Abstract-results part. “According to both models, the 40 GW 2050 target for ocean energy set by the European Commission in 2020 could be reached if European coastal countries, including associated countries to the EU-27, adopt supportive policies for both technologies immediately.” The associated countries include Norway and the UK.  CB 2.1: 1 ^st^ paragraph: wave and tidal are the most advanced ocean energy technologies, next to offshore wind, better to expand the wording for inclusion of offshore wind. More literature references for the wave and tidal potential may be helpful. Gunn and Stock-Williams (2012 ^1^ ) find up to 18,500 TWh and Mork et al. find up to 26,100 TWh ( https://doi.org/10.1115/OMAE2010-20473). Nevertheless, the study is on Europe, thus, the potential for wave power and tidal power in Europe would be of particular interest. You may also mention the potential of offshore wind, so that the three technologies you focus on in the paper are all presented with their potential in Europe.

BL 2.1: We normally use the definition of Ocean Energy Europe to define wave, tidal, salinity gradient & OTEC as Ocean Energy. Offshore wind and offshore solar are separate in this definition. To avoid any misunderstanding we are differentiating between “Offshore Renewable Energy” and “Ocean Energy” throughout the paper. We also added the additional references and split up the potential in all three sources. We stayed with the global potential as the sources for Europe are not fully conclusive on the total potential.  CB 2.2: 2 ^nd^ paragraph: better to clarify the targets for ocean energy in addition to offshore wind, as the latter may have separate targets, given the numbers presented. BL 2.2: Thank you for pointing this out. We added the data and changed the flow of the paragraph to explain why we are explaining LCoE and capacity at all. We also added the offshore wind targets for consistency and in relationship to Christian’s comment.  CB 2.3 4 ^th^ paragraph: it may be worth mentioning whether in other parts in the world, in particular in Asia, one can observe progress in the deployment of wave and tidal power. Experience shows that a major breakthrough in other world regions may induce strong development in other regions as well. Please check the 4 ^th^ sentence for the grammar. BL 2.3: We added the proposed description on funding mechanisms in the EU, UK and US. We also added Asian countries with a strong dedication for offshore renewable energy. We also improved the writing of the paragraph. CB 2.4: It would be interesting to add one paragraph on literature on the role of wave and tidal power for Europe. Are the two technologies mentioned in WEO reports of the IEA for Europe? Is there scientific literature on the role of the two technologies, for Europe, or for single countries? For the IEA WEO I checked it but only global numbers are reported in recent reports, no numbers for Europe. On a country basis at least a recent study for the UK, which found up to 42 GW installed capacity in the UK and Ireland until 2050 (Alvarez-Silva et al., 2016 ^3^ ), this may be valuable for this research as it documents a value add of wave power for the energy transition from an energy system perspective. In the energy [r]evolution scenario of Greenpeace one can find 53 GW of ocean energy installed in OECD Europe by 2050 ( https://www.greenpeace.org/static/planet4-canada-stateless/2018/06/Energy-Revolution-2015-Full.pdf; Tab. 13.4.18), which may be a valuable reference, given the low coverage of ocean energy in energy scenarios. More related research may be quite helpful for the aims of the paper.. BL 2.4: We addressed the novelty comment from Shona and Energy system from Christian together, referring to current negligence on wave and tidal in most energy system reports. Additionally, 25 references are added of which 11 are from scientific journals.  CB 3.6: There is an unexpected break in the flow from the 2nd to the 3rd paragraph (from wind to wave/tidal). Having the section headline expanded to ‘Starting values and years for wave and tidal’ may ease that a bit. BL 3.6: Great suggestion, we adopted that change.  CB 3.7: p. 4, 1st paragraph: ‘power purchase agreement’ is used again as a word, while the abbreviation PPA was introduced earlier in this section. Another example is CAGR what’s again used in the long version on the next page, while the abbreviation was introduced before. Please check for all abbreviations that they are used consequently, once the abbreviation was introduced. p. 5 (and others): please check all used abbreviations whether they are introduced. For instance OEE and ORE appears, while this should be abbreviations. BL 3.6: Thanks for pointing out those inconsistencies, we adopted that change.  CB 3.8: Table 1: Sweden is abbreviated with ‘SW’, while the typical convention should be ‘SE’, please check for the entire manuscript. p. 4 right column, 3rd paragraph: ‘bad correlation’ may be better as 'poor' or 'weak' correlation. BL 3.8: We adopted those changes.  CB 3.9: p. 5, 1st paragraph: three exponential functions are mentioned. It may be helpful to mention the three phases so that it is explicitly clear to what ‘three’ refers. BL 3.9: We added numbering to make the link clearer.   CB 3.10: p. 5/6: section on LCoE is important for the entire paper. Please introduce the formula for LCoE. BL 3.10: The fit to the LCoE formula was added insterad of the formula as no direct LCoE calculation was done by us.  CB 3.11: p. 6 left column bottom: “… the values given in the table.” Which table? Please refer explicitly to the table referred to in the paper. p. 6: Tab. 2 is not referenced in the text, please embed it into the text. Where are the starting values for tidal and wave LCoE for 1 MW from? These are among the most important references, as the derived costs are directly linked to these starting values. Please discuss the validity of these starting values, as they have a very strong impact on the results. You may consider a sensitivity for these values. The link of LCoE to the capacity remains unclear. What formula links LCoE to the capacities? Is a learning rate applied? If so, which value(s) and based on what reference? The Tab. 2 seems to be central, while the impact of the capacity on the LCoE remains unclear. BL 3.11: We added the reference to table 2 and explicitly referred to the table as indicated above. The table as well as text now includes the calculated learning rates from the OEE values. The numbers by OEE are not supported by specific learning rates but the table contains the values re-evaluated from the provided table. This should provide more clarity on the source as well as the general meaning of the values.  CB 4.3: p. 8, 3rd paragraph: it may be worth checking additional literature for the wave power potential of 95 GW in Europe. There is unpublished research with much higher values (more than an order of magnitude more, however, for entire Europe, thus, explicitly including the UK and Norway), therefore, other literature may also find higher values. Please also clarify for the wave power potential for Europe, whether entire Europe (incl. the UK, Norway, Iceland, etc.) is meant, or EU-27. For the case of ocean energy this makes a considerable difference. The best may be to clarify across the entire paper whether EU-27 or entire Europe is meant, whenever the term ‘Europe’ is used. In the right column it is mentioned that the UK is assumed to contribute to the EU targets. That most important assumption is better mentioned earlier in the paper, when the term ‘Europe’ is used for the first time in the context of this paper. p. 8, section European targets: please add per additionally mentioned category (salinity gradient, OTEC, floating PV) at least one scientific reference. Good literature may be (please check for yourself): Alvarez-Silva et al. (20163); Langer et al. (20204); Shi et al. (20235); Breyer et al. (20236); however, at least OTEC seems not to be that attractive for Europe given the tropical water temperature required, while the Mediterranean may develop towards that directly due to climate change, unfortunately. BL 4.3: The reference of Mork et al was added to increase the range of values provided. Satymov et al. does not include a regional breakdown of energy potential for European regions and was not added. We also tried to clarify better throughout the paper which “Europe” we are referring to (EU-27 vs EU+associated countries) - see comment 1.1.   CB 4.4: p. 8, Fig. 5: link to the (new) methods how the LCoE decline over time. What is the learning rate? How is the cost decline calculated? It’s not clear in the paper how this is calculated. BL 4.4: See comment 3.11 - Based on the values for LCOEs published by OEE the learning rates have been re-calculated. A table on these values has been added showing also these re-calculated LR values.   CB 4.5: p. 10, 1st paragraph: ‘deployment’ may be better suited then ‘journey’. p. 10, section offshore wind: it may be helpful to better explain what ‘MetOcean’ shall be. BL 4.5: We implemented those changes and replaced MetOcean with offshore measurement campaigns.  CB 4.6: p. 10, section ‘Best case scenario’. It may be helpful to introduce the ‘Best case scenario’ already in the Methods section, so that readers are prepared on different scenarios or respective variations. That may be done best in a paragraph on scenarios to be considered. BL 4.5: We added a subsection to the methods that introduces all considered scenarios CB 5.2: p. 10, 1st paragraph of the section. The term ‘net zero emission’ links nicely to the same from the Introduction. It may be helpful to expand the section on ‘net zero emissions’ a bit as this is clearly set in the European Green Deal. Different pathways are possible to reach that aim, while highly renewable energy system transition options may be most likely, also cost-wise. This and a review on comparable studies was published here (Breyer et al., 20227), where wave and tidal power is missing in almost all studies, and that research gap may be documented with this overview, while the previously mentioned paper on the UK clearly indicates that latest research may be able to close this gap. In that regard, it may be helpful that at least for three European islands wave and tidal power systems had been investigated as part of a highly renewable energy system and published in scientific articles (Meschede et al., 20228; see Tab. 5), as detected for the cases of El Hierro, Faroe, and Sardinia. Not sure whether this may be a helpful indication of upcoming wave and tidal power systems for the benefit of the paper, as island energy systems. You may mention, that wave and tidal power could also develop faster than wind offshore, since many challenges (such as grid connection, O&M, harbour logistics, etc.) have been fixed for wind offshore, thus, wave and tidal power can build on that, which should enable a faster growth potential. BL 5.1: We added additional publications and expanded upon this in the discussion section.  Apologies again for the delay in our reply and we reamin to further improve parts of the paper if needed. Thanks a lot for reviewing this work and helping us improving it.

# Reviewer report: Reviewer response for version 1

**DOI:** 10.21956/openreseurope.17180.r33213

**Published:** 2023-07-13

**Authors:** Shona Pennock

**Affiliations:** 1 School of Engineering, The University of Edinburgh, Edinburgh, UK

This paper details an analysis of the potential deployment and cost reduction trajectories for wave and tidal technologies, based on historical wind deployment trajectories and industry-recognised ocean energy cost reduction trajectories from Ocean Energy Europe. This is important and interesting work, and was a pleasure to read. The paper is well structured, easy to read and understand, and the figures are also clear and well presented.

 As a general comment, this paper could do with a proof read by a native english speaker for grammar and wording choice. There are some small errors throughout, such as prepositions out of place. To name one example, the third paragraph should read 'By the beginning..' and not 'By beginning'. These small errors do not affect the reader's ability to understand the work, however.

 Some further suggestions for more specific changes and improvements are below:

**Introduction:**

- When discussing the potential LCoE of wave and tidal in the introduction, it could be useful to frame this in context, e.g. of the current LCoE of offshore wind, or compared to average day-ahead electricity prices over the last decade. A reader with little knowledge of these might stuggle to understand the importance of further cost reduction for ocean energy. Similarly, it might also be useful to reference the current estimated LCoE of wave and tidal in the introduction section, to give some initial context.
- In the introduction you state that there have been positive policy developments in the EU, US, and UK. It would be good to give a couple of brief examples of these for the reader, e.g. the millions spent in innovation funding for ocean energy through Horizon Europe, the market-pull support schemes in the UK including the Renewables Obligation and Feed-in Tarriffs with Contracts for Difference.
- There needs to be some words in the introduction to explain the novelty of this work, and what it adds to the existing literature. I'd also recommend that some more scientific journal papers are referenced at this point.

**Methods:**

- For repeatability, the offshore wind database used for this analysis needs to be referenced in the first paragraph of the methods section. The ocean energy europe database should also be referenced in the third paragraph.
- It would add some clarity for the reader to have some kind of flow chart, or numbered list to explain the key steps of the methodology, before explaining each stage further in the sub-sections.
- In equation 1 I think the Pcum,0 should be replaced by Py,0 - if I have understood this correctly.
- For equation 2, I was unsure why 'y' is being used to denote the capacity figure, when 'P' is used to denote installed capacity in equation 1. From the explanatory text it reads as if the nomeclature is inconsistent between these two equations. It also seems that 'q' is then being used for capacity in equations 3-6.
- It seems inconsistent that five-year intervals were chosen for the doubling time model, when ten-year intervals were chosen for the growth model. Some further explanation is needed to justify this.
- In figure 1, 'a', 'b' and 'N' should be defined in the figure caption so that a reader can understand the figure more easily at a glance.

**Results:**

- It would be clearer in figures 1, 2 and 3, to label the two separate graphs as (a) and (b), rather than (1) and (2), so as not to confuse with the figure number. Likewise with figures 6, 7, and 9.
- In the caption for figure 4, it would be useful to specify that this is the percentage of the targets fulfilled specifically by wave and tidal stream technologies.
- In figure 6, has this sensitivity been undertaken for the wave/tidal results, or the more general model, extrapolating from the offshore wind figures? It would be useful to have some text to discuss how this sensitivity analysis would be reflected in the wave and tidal technology results (e.g. by how much would the 2030 LCOE targets be delayed for each of these sensitivity cases).

**Discussion/conclusion:**

- Another key assumption that is worth discussing is the cost reduction trajectory for wave and tidal technologies. While this is based on the best current estimations of the sector, as presented by Ocean Energy Europe, there will be a great deal of uncertainty associated with these due to the early development stage of the sector. This cost reduction could also be very technology specific, for example to different wave or tidal device designs. The ability to achieve this cost reduction is also highly sensitive to the available policy support, such as innovation funding.

Is the study design appropriate and does the work have academic merit?

Yes

Is the work clearly and accurately presented and does it cite the current literature?

Partly

If applicable, is the statistical analysis and its interpretation appropriate?

Not applicable

Are all the source data underlying the results available to ensure full reproducibility?

Yes

Are the conclusions drawn adequately supported by the results?

Yes

Are sufficient details of methods and analysis provided to allow replication by others?

Partly

Reviewer Expertise:

Economics of innovation, ocean energy techno-economics, ocean energy technologies

I confirm that I have read this submission and believe that I have an appropriate level of expertise to confirm that it is of an acceptable scientific standard, however I have significant reservations, as outlined above.

# Response

**Published:** 2024-08-08

**Authors:** Benjamin Lehner

Thank you Shona for this detailed and valuable feedback and excuses for the delayed reply. We will upload the latest version in the coming days but please find below already our answers to your remarks ( *in cursive)* one by one with the replies underlined. 

 SP 2.2:When discussing the potential LCoE of wave and tidal in the introduction, it could be useful to frame this in context, e.g. of the current LCoE of offshore wind, or compared to average day-ahead electricity prices over the last decade. A reader with little knowledge of these might struggle to understand the importance of further cost reduction for ocean energy. Similarly, it might also be useful to reference the current estimated LCoE of wave and tidal in the introduction section, to give some initial context.

BL 2.2: Thank you for pointing this out. We added the data and changed the flow of the paragraph to explain why we are explaining LCoE and capacity at all. We also added the offshore wind targets for consistency and in relationship to Christian’s comment.

 SP 2.3 In the introduction you state that there have been positive policy developments in the EU, US, and UK. It would be good to give a couple of brief examples of these for the reader, e.g. the millions spent in innovation funding for ocean energy through Horizon Europe, the market-pull support schemes in the UK including the Renewables Obligation and Feed-in Tariffs with Contracts for Difference. BL 2.3: We added the proposed description on funding mechanisms in the EU, UK and US. We also added Asian countries with a strong dedication for offshore renewable energy. We also improved the writing of the paragraph. SP 2.4: There needs to be some words in the introduction to explain the novelty of this work, and what it adds to the existing literature. I'd also recommend that some more scientific journal papers are referenced at this point.

 5 ^th^ paragraph: ‘forecast’ typically describes periods up to 5 not more than 10 years, while longer periods in the order of decades may be better described by ‘projection’ for reflecting a higher degree of uncertainty.

BL 2.4: We addressed the novelty comment from Shona and Energy system from Christian together, referring to current negligence on wave and tidal in most energy system reports. Additionally, 25 references are added of which 11 are from scientific journals.  SP 3.1: For repeatability, the offshore wind database used for this analysis needs to be referenced in the first paragraph of the methods section. The ocean energy europe database should also be referenced in the third paragraph.

BL 3.1: We added the offshore wind database but sadly do not have permission to make the data from OEE public.    SP 3.2 It would add some clarity for the reader to have some kind of flow chart, or numbered list to explain the key steps of the methodology, before explaining each stage further in the sub-sections. BL 3.2: We agree with the reviewer. A flow chart summarizing the key steps taken to build the presented model  is added to the beginning of the methods section. Each step is then further elaborated in sub-sections. Key steps methodology added:

1. Data acquisition on bottom-fixed offshore wind farms and setting the reference year.
2. Data acquisition of wave and tidal projects in Europe
  1. filtering out disappeared and prolonged hibernating projects
  2. Determine the pre-commercial start years for wave and tidal
3. Fit growth curve based on the installed cumulative bottom-fixed offshore wind 
  1. Exponential fit based on cumulative capacity
    1. Three different phases: lag, kickoff and growth
  2. Doubling time curve fit
    1. five different phases
4. Apply growth curves to wave and tidal
5. LCOE forecast based on predicted wave and tidal growth

  SP 3.3 In equation 1 I think the Pcum,0 should be replaced by Py,0 - if I have understood this correctly. For equation 2, I was unsure why 'y' is being used to denote the capacity figure, when 'P' is used to denote installed capacity in equation 1. From the explanatory text it reads as if the nomenclature is inconsistent between these two equations. It also seems that 'q' is then being used for capacity in equations 3-6. BL 3.3: We changed “Pcum,0” by “Py,0” in the text. Therefore, “Py” stands for the cumulative capacity at the year “y”. We also replaced “q” by “P” in equations 3 to 6 to stay consistent in the nomenclature. Regarding equation 2, we used “y” and not “P” because it is a general formula which can be applied to numerous fields. SP 3.4 It seems inconsistent that five-year intervals were chosen for the doubling time model, when ten-year intervals were chosen for the growth model. Some further explanation is needed to justify this. BL 3.4:  Regarding the growth model, we wanted to show that there were three different phases during the development of the offshore wind sector: the lag phase with only few pilot projects, the kick-off phase with the commissioning of the first commercial farms and the growth phase with the quick expansion of the offshore industry. We wanted to apply those three different phases to the tidal energy and wave energy industry. Regarding the doubling time model, we tried to find the best balance between a high coefficient of determination and a good correlation with the data points. After trying different intervals, it appeared that a 5-year interval was the best compromise. SP 3.5 In figure 1, 'a', 'b' and 'N' should be defined in the figure caption so that a reader can understand the figure more easily at a glance. BL 3.5: We added those definitions to the figure caption.    SP 4.1: It would be clearer in figures 1, 2 and 3, to label the two separate graphs as (a) and (b), rather than (1) and (2), so as not to confuse with the figure number. Likewise with figures 6, 7, and 9. In the caption for figure 4, it would be useful to specify that this is the percentage of the targets fulfilled specifically by wave and tidal stream technologies. BL 4.1: We added those changes.    SP 4.2: In figure 6, has this sensitivity been undertaken for the wave/tidal results, or the more general model, extrapolating from the offshore wind figures? It would be useful to have some text to discuss how this sensitivity analysis would be reflected in the wave and tidal technology results (e.g. by how much would the 2030 LCOE targets be delayed for each of these sensitivity cases). BL 4.2: This sensitivity analysis has only been applied to the starting capacity and/or starting year of wave and tidal, as their starting value and year are not clearly identifiable, both being in a pre-commercial stage. No sensitivity run on the reference year/capacity of offshore wind has been performed, as its starting year and capacity is more identifiable (the first offshore wind array commissioned).    SP 5.1:  Another key assumption that is worth discussing is the cost reduction trajectory for wave and tidal technologies. While this is based on the best current estimations of the sector, as presented by Ocean Energy Europe, there will be a great deal of uncertainty associated with these due to the early development stage of the sector. This cost reduction could also be very technology specific, for example to different wave or tidal device designs. The ability to achieve this cost reduction is also highly sensitive to the available policy support, such as innovation funding.  BL 5.1: Most of the data here is based on OEE and an EU-SCORES report we did on this topic ( https://euscores.eu/wp-content/uploads/2022/11/D.7.9-LCOE-Analysis-for-baseline-V3_07.10.2022.pdf) . We are also planning to launch a new report from EU-SCORES soon to further advance this work

   Apologies again for the delay in our reply and we reamin to further improve parts of the paper if needed. Thanks a lot for reviewing this work and helping us improving it.
